# Inter-rater Reliability of a 13-Category Arterial Doppler Waveform Classification and Practice of French Vascular Physicians

**DOI:** 10.3389/fcvm.2021.640838

**Published:** 2021-05-25

**Authors:** Damien Lanéelle, Jérôme Guillaumat, Jean-Eudes Trihan, Camille Pottier, Loukman Omarjee, Guillaume Mahé, SFMV PAD Study Group

**Affiliations:** ^1^Department of Vascular Medicine, CHU Caen Normandie, Caen, France; ^2^Université de Caen Normandie, INSERM, COMETE 1075, Caen, France; ^3^Department of Vascular Medicine, CHU Poitiers, Poitiers, France; ^4^Université de Rennes 1, INSERM CIC 1414, Rennes, France; ^5^Department of Vascular Medicine, CHU Rennes, Rennes, France

**Keywords:** Doppler spectrum, Doppler waveform analysis, periphera arterial disease, Doppler waveform classification, vascular diagnosis

## Abstract

**Background:** Arterial Doppler Ultrasound waveform (DW) analysis allows the detection and evaluation of lower extremity peripheral artery disease. The high heterogeneity of the reported description of DW is reduced by the use of classification. However, the reliability of these classifications is either unknown or low to moderate and practices of vascular caregivers regarding the use of these classifications remain unknown.

**Aims:** This study aims to assess the inter-observer reliability of the Saint-Bonnet classification, a 13-category DW classification. The secondary objective was to determine the utilization rate of the most common classifications and the ability of these classifications to describe DW.

**Methods:** A national survey was conducted among all vascular physicians of French society of vascular medicine. They were invited by email to describe on a website 20 DW without and with the display of the Saint-Bonnet classification. The reliability of this classification was estimated by Fleiss' Kappa expressed with [95% confidence interval]. A semantic analysis allowed us to classify the physicians' responses according to the terms used. Finally we have evaluated for each classification the rate of misuse, i.e., the addition of a complementary term to the defined categories.

**Results:** One hundred and ten physicians participated and only 5% of these were familiar with Saint-Bonnet classification. Fifty-four percent of vascular physicians used no classification at all. Vascular physicians used the Spronk (four-category), Descotes (five-category) and Saint-Bonnet (13-category) classifications for respectively, 31, 10, and 5%. Kappa coefficient of Fleiss (κ) was 0.546 [0.544–0.547] (*p* < 0.001). Reliability by category ranges from κ of 0.075 to 0.864. In multivariate analysis, the use of a classification was associated with fewer years of experience and was dependent on geographic location. Misuse rate by classification was 88, 82, and 5% using Spronk, Descotes and Saint-Bonnet classifications respectively.

**Conclusion:** The reliability of Saint-Bonnet classification is weak to moderate by vascular physicians who are not familiar with its use. However, unlike the other classifications, it seems to be sufficiently precise so that the user does not need to complete its description. There is a significant heterogeneity in the use of arterial Doppler classifications in France.

## Background

Arterial Doppler ultrasound is commonly used for non-invasive diagnosis of peripheral artery disease (PAD) (1) and analysis of Doppler ultrasound waveforms (DW) is a proficient method for evaluating the disease severity (2). Indeed, the aspect of the DW at rest, below an arterial lesion is the hemodynamic result of several components: the vessel wall compliance, the direct effect of the stenosis or occlusion, and the distal vasodilatation level. There is significant heterogeneity in the DW description, leading to confusion among care providers but which can be significantly reduced by the use of a DW classification (3–5). Different classifications have been proposed, some with four category (6), others with 5 (7) or 13-category (8) but reliability of this DW classification is either unknown or weak to moderate for the one with the fewest categories as shown among American and Chinese care givers (9, 10). Furthermore, the practices of caregivers performing ultrasounds remain unknown with respect to the use of the three main classifications [Spronk classification (four-category), Descotes (five-category) and Saint-Bonnet (13-category)] in France.

This study aims to assess the inter-observer reliability of the 13-category DW classification. The secondary objective was to determine the utilization rate of the most common classifications. In addition, there appears to be a misuse of classifications with few categories, prompting physicians to add one or more terms to the initial categories to improve the description. This misuse, contrary to the initial purpose of standardization and reduction of variability, has never been described or quantified and is also the subject of this work.

## Methods

All vascular physicians who are members of French society of vascular medicine (SFMV) were invited to participate by email. This study was validated by the ethical committee of Rennes, France (n° 1818) and registered on clinicaltrials.gov (NCT03827512).

A three-step online questionnaire based on arterial DW record (Supplementary Material) was presented on the website of the SFMV. The first part consisted of general questions: the localization and years of practice, the practitioner's age and mode of practice (hospital, private practice, mixed activity). The second part of the questionnaire presented 20 DW with a free text area to describe in a few words the aspect of the DW as it would be described in a medical report. The arterial flows were presented with the usual settings (scrolling speed at 66 mm/s) without precision on the recording conditions (insonation angle, gate width, and probe type) considered as optimal. The heterogeneity of descriptions was defined by the number of different descriptions for the same DW. The third part of the questionnaire assessed the inter-observer reliability of the 13-category classification. The same DWs that were presented in part 2 were shown but this time with the display of a 13-category classification (Saint-Bonnet). In this part, physicians were asked to describe DW using the Saint-Bonnet classification. The total response time to this questionnaire was 15–20 min.

We previously selected the DW to cover all possible situations encountered in common clinical practice. A semantic analysis allowed us to classify the physicians' responses according to the terms used. Three different classifications were identified from the responses to describe the waveform: Spronk (4-category), Descotes (5-category) and Saint-Bonnet (13-category). In the free interpretation, the Saint-Bonnet classification could be used as well as the others, whereas in the third part of the questionnaire, only the Saint-Bonnet classification had to be used by all the participants.

Classification by Spronk is comprised of four waveform categories and described as follows in the original publication (6):

– Triphasic: three phases—forward flow, flow reversal, and a second forward component
– Biphasic: two phases—one forward flow and one reverse component
– Monophasic: single phase—forward flow with no reverse flow component
– Other: when the DW does not correspond to any previous category.

Classification by Descotes was proposed in 1975 and suggests five types in diagnosing an arterial obstruction. The various types (from type 0 or N to type 4) are described as follows in the original publication (7):

- Type 0 or N: normal profile
- Type 1: Disappearance of the reflux wave and the second positive wave
- Type 2: lengthening of the time of descent of the positive wave
- Type 3: lengthening of the time of descent and ascent, disappearance of the interval between 2 successive waves
- Type 4: severe deterioration preceding the occlusion, very low speed, difficult to record.

The simplified Saint-Bonnet classification was proposed in 2017, comprises eight types, and allows considering the continuous character of the flow (mentioned with the abbreviation CF) for 13 total categories. The various types are described as follows in the original publication (8, 11):

- Stage N: short ascent time, short descent time, the negative diastolic component, the positive diastolic rebound and the return to baseline
- Stage A: the disappearance of positive diastolic rebound
- Stage B: the disappearance of the negative diastolic component
- Stage CD: Increase in fall time and systolic rise time with the presence of a “blunted” systolic peak
- Stage E: Loss of signal phase
- Stage 0: No waveform
- Stage FA: short rise time and short fall time followed by a long diastolic negative component (False aneurysm DW),
- Stage U: when the DW does not correspond to any previous category.

If the signal does not return to the baseline, the flow is said to be continuous (CF) and can be encountered at all stages of classification (except for the Saint-Bonnet 0, U and FA stages).

We have also differentiated, by response, the use of the classification in its original form or the addition of a detailed information (adverb, precision, object complement).

We considered that the caregiver used the Spronk classification if their descriptions contained “monophasic,” “biphasic” “triphasic” and “sharp,” “poor” or “blunted” to characterize the waveforms. We considered that the caregiver used the Descotes classification if their responses contained from type 0 to type 4. We considered that the caregiver used the Saint-Bonnet Classification if their responses contained from type A to type E and type U, FA, 0 and the mention CF. A physician was considered to be a user of a classification when 50% of his or her responses were related to it.

The use of a classification was considered to deviate from its original form if it contained other elements of description like the use of an adverb, adjective or other detailed information (good, satisfactory, correct, fair, average, moderate, extensive, bad, slightly, little, enough, partially, clearly, discreet, damped, tardus, delayed, parvus, attenuated, parvus et tardus, rebound, notch, ample, resistive, non-laminar, dispersion, turbulent, disordered, chaotic).

### Statistical Analysis

Results are expressed in mean ± standard deviation or as a percentage. When the mean does not allow the expression of the dispersion of the quantitative data, they are expressed as a median with [third quartile − first quartile = InterQuartile Range IQR]. Wilcoxon-test was used to compare the heterogeneity of descriptions between the free interpretation and that with suggested Saint-Bonnet classification. We calculated inter-rater reliability between the physicians for the arterial DW classification through the Kappa coefficient of Fleiss (κ). The reliability of this classification was estimated by Fleiss' Kappa expressed with [95% confidence interval]. Two different interpretations of kappa values were used: that of Landis and Koch, which is the most used (12) and that of McHugh (13), which is more stringent. The Landis and Koch interpretation: 0.21–0.40: fair; 0.41–0.60: moderate, 0.61–0.80: substantial; >0.80: almost perfect. The McHugh interpretation: 0.21–0.39: minimal; 0.40–0.59: weak; 0.60–0.79: moderate; 0.80–0.90: strong; >0.90: almost perfect. We performed the analysis using R software version 3.0.1 (R Foundation for Statistical Computing, Vienna, Austria; www.R-project.org), with the package “irr” (14). Multivariable logistic regression analyses after univariate feature selection (forward stepwise) were used to identify the factors explaining the differences in the use of classification by physicians. A *p*-value < 0.05 was considered as statistically significant.

## Results

### Participation

Of the 1936 solicitations sent out, 380 vascular physicians (19.6%) responded and 110 of those responses (5.6%) were complete and analyzable. These 110 vascular physicians had a hospital activity in 38% of cases, a private practice in 48% and a mixed activity in 14%. They were from different parts of France with 1–48 years of ultrasound experience. Median number of years working in vascular ultrasound was 13 years [24−5=19 IQR]. The characteristics of the population are presented in Table 1.

**Table 1:** Epidemiological characteristics of French vascular physicians.

**Variables**	**Vascular physicians (*n* = 110)**
Age (year ± sd)	45 ± 11
Ultrasound experience (year ± sd)	15 ± 10
Male sex (%)	71%
Hospital Activity (%)	40%
Secondary hospital (%)	15%
Tertiary hospital (University Hospital) (%)	25%
Private practice or mixed activity (%)	60%
Worked alone (%)	23%
Group practice (%)	26%
Private clinic (%)	8%
Other (%)	3%

*sd, standard deviation*.

### Practice of French Vascular Physicians

**Figure 1:**
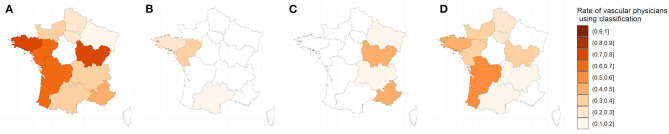
Map of the use of classifications by vascular physicians. **(A)** One of the three classifications. **(B)** Saint-Bonnet Classification. **(C)** Descotes classification. **(D)**. Spronk classification.

The semantic analysis of the spontaneous responses shows that 54% percent of vascular physicians used no classification at all. They used Spronk (4-category) classification for 31% of them, the Descotes (5-category) classification for 10% and the classification of Saint-Bonnet (13-category) in 5% (Figure 1).

For the Descotes, Spronk and Saint-Bonnet classifications, supplemental information was given in 82, 88, and 5%, respectively (*p* < 0.005).

In univariate analysis, there is a significant difference in the use of classification between vascular physicians according to the age, year of experience, practice activity and localization but no difference according to the gender. The multivariate analysis showed that only experience (the use of a classification is inversely proportional to the age of exercise) and localization (the use of a classification is associated with region of exercise) had a significant influence on the use or not of either classification (Table 2).

**Table 2:** Effect of age, gender, activity type, geographic location, and experience on the use of a classification.

**Physician characteristics**	**Univariate analysis**		**Multivariate analysis**	
	**OR (95% CI)**	***p*-value**	**OR (95% CI)**	***p*-value**
Gender		0.092	–	
Male	Reference		Reference	
Female	0.49 (0.21–1.12)	0.097	–	
Age (in years)	0.94 (0.91–0.98)	0.002	–	
Experience (in years)	0.93 (0.89–0.97)	0.002	0.94 (0.89–0.98)	0.003
Geographic location		0.040		0.021
Practice activity		0.023		0.104
Private	Reference		Reference	
Hospital	2.54 (1.14–5.84)	0.025	1.36 (0.50–3.67)	0.104

*OR, Odds Ratio; CI, Confidence Interval. The variables “age” and “experience” being collinear, we have only included in the multivariate model the variable with the highest significance (experience). In order to keep the table readable, only the overall effect of geographic location is shown without the odd ratio per region*.

### Inter-rater Reliability of Saint-Bonnet Classification

**Figure 2:**
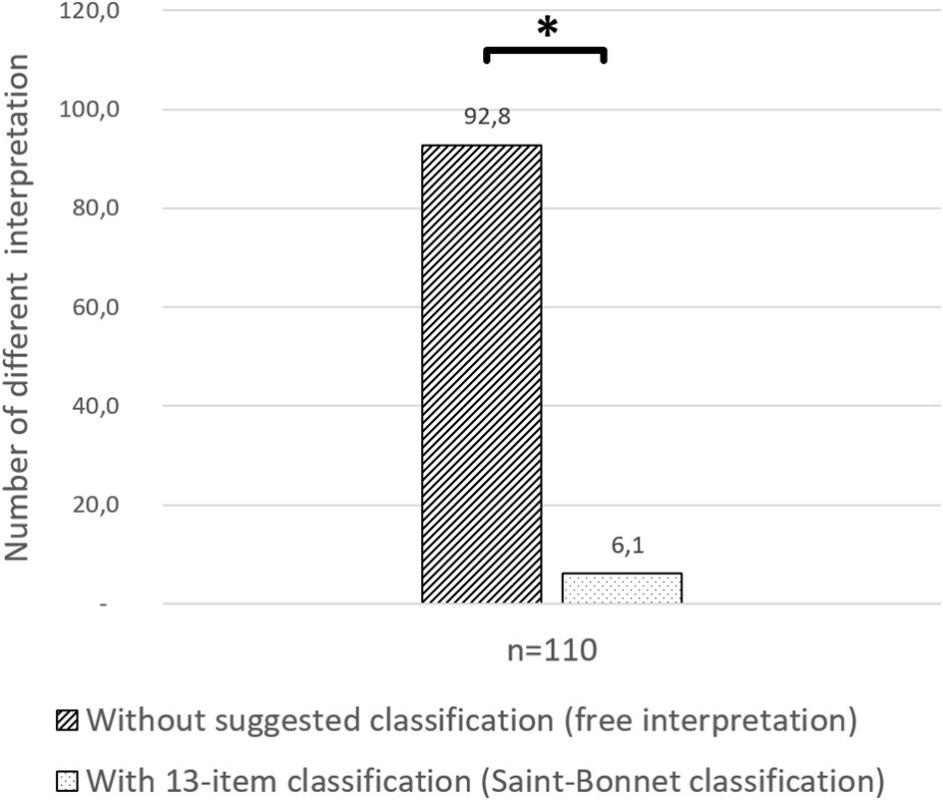
Heterogeneity comparison. Number of different responses of the 20 Doppler waveforms among 110 participants with free interpretation and with suggested Saint-Bonnet classification. *p < 0.05.

Fleiss' Kappa (κ) for 110 French vascular physicians using Saint-Bonnet classification concerning 20 DW was 0.546 [0.544–0.547] (*p* < 0.001). Inter-rate reliability among categories varies between κ of 0.075 and 0.864 and is shown in Table 3. The average heterogeneity in case of free interpretation is 92.8 ± 10.7. The use of the Saint-Bonnet classification allows to reduce the average heterogeneity to 6.1 ± 2 (Figure 2).

**Table 3:** Inter rater reliability of arterial Doppler waveform classification by categories from 110 raters and 20 Doppler waveforms.

**Category**	**Kappa [95% CI]**	***p*-value**
N	0.617 [0.616–0.619]	<0.0001
N-CF	0.434 [0.429–0.439]	<0.0001
A	0.864 [0.862–0.867]	<0.0001
A-CF	0.387 [0.384–0.390]	<0.0001
B	0.586 [0.585–0.587]	<0.0001
B-CF	0.243 [0.240–0.247]	<0.0001
CD	0.460 [0.455–0.566]	<0.0001
CD-CF	0.622 [0.620–0.624]	<0.0001
E	0.463 [0.459–0.566]	<0.0001
E-CF	0.702 [0.701–0.703]	<0.0001
FA	0.621 [0.620–0.623]	<0.0001
U	0.075 [0.055–0.096]	<0.0001
Global Doppler classification	0.546 [0.544–0.547]	<0.0001

*N, short ascent time, short descent time, the negative diastolic component, the positive diastolic rebound and the return to baseline; A, disappearance of positive diastolic rebound; B, disappearance of the negative diastolic component; CD, Increase in fall time and systolic rise time with the presence of a “blunted” systolic peak; E, Loss of signal phase; FA, short rise time and short fall time followed by a long diastolic negative component; U, when the DW does not correspond to any previous category; CF, when the signal does not return to the baseline. CI, confidence interval*.

## Discussion

This is the first study assessing the reliability of 13-category Saint-Bonnet classification in a whole country. It has already been noted the importance of the use of a any classification to reduce the heterogeneity of DW descriptions and this work confirms previous data obtained with other classifications and in other countries (3–5). However, the classification should not be too restrictive to avoid the risk of not being discriminatory (with only two categories at the extreme: normal and abnormal). Conversely, excessive multiplication of categories can lead to confusion and possible assignment of a DW to two different categories.

This Saint-Bonnet classification is the classification taught in France for the past 2 years for the vascular residents but which is not part of the continuing education program for practicing physicians (8). It contains 13 categories that allow a better classification of the DW than other classification (11, 15). In the present study, the reliability is considered as weak to moderate (κ = 0.546 [0.544–0.547], *p* < 0.005) in vascular caregivers unfamiliar with this Saint-Bonnet classification. Reliability of a four-category classification has been studied among Chinese and American populations and the inter-rater reliability is minimal to moderate, κ ranged from 0.402 to 0.522 (9, 10). It can be noted that, in spite of a large number of choices, the Saint-Bonnet classification does not seem less reliable than a four-category classification. The “U” category obtained the worst result (κ = 0.075) with a zero reliability, although reliabilities of the “A” or “E-CF” categories were strong (respectively κ = 0.864 [0.861–0.892] and κ = 0.702 [0.698–0.707]). It is possible that the use of the “U” category, which corresponds to a DW that cannot be classified in any other category and therefore does not correspond to any well-established hemodynamic pattern, does not bring any benefit with respect to the initial purpose: standardization and reduction of the heterogeneity of the DW descriptions. This “weak” or “moderate” inter-rater reliability should be regarded with caution since the vascular physicians were using this 13 category Saint-Bonnet classification for the first time and were therefore unfamiliar with it. Indeed, only 5% were familiar with the Saint-bonnet classification. The explanation is that this classification was recently adopted by the French Vascular Medicine teachers. Unfortunately it was not possible to assess the reliability of the classification in this small subgroup of physicians who were familiar with the saint-Bonnet classification. We would expect a better reliability in physicians or sonographers familiar with this classification. This remains to be studied.

This study also highlights the practices and the description of DW by French vascular physicians of which 54% do not use any classification, 31% use Spronk and 10% use Descotes. The lesser use of the Saint-Bonnet classification is probably related to its more recent description (2017) compared to that of Descotes and Spronck (1975 and 2005, respectively). Nevertheless, these “old” classifications are not used “as there were initially described” and physicians almost systematically (88% for Spronk and 82% for Descotes) add one or more terms to the category to characterize the DW description (example: slightly damped biphasic, continuous monophasic, type 2 resistive). This misuse, contrary to the initial objective of the classification, increases the heterogeneity of the DW descriptions. The Saint-Bonnet classification does not seem to be subject to this misuse since very few (5%) complementary terms are added to the category by physicians, making it a classification of choice for standardizing DW descriptions but this should be confirmed in a larger sample of users.

Of interest we also assessed the reasons of the variability of the use of the classifications. The geographical variability in the use of classification is probably related to the habits of training locations. These results confirm that the description of DW is an issue in the vascular field and this is a barrier to the homogeneity of vascular analyses and the use of Doppler ultrasound in common vascular explorations as well as in clinical research. Regarding the use of a classification based on experience, it should be noted that in previous decades, medicine was less standardized and recommendations were rarer and less disseminated. We can therefore hypothesize that older practitioners have not been trained in the use of DW classifications. Another hypothesis is that with experience comes the conviction that accurate DW classification, although allowing standardization of practice, is of little clinical interest. This is the first time that such data about the use of Doppler waveforms is published demonstrating the issue of the homogenization of the teaching on a whole country. Similar issues were found for ABI and pressures measurements in France (16, 17).

Furthermore, the 13-category classification of Saint-Bonnet is used by only 5% of the participants, which is expected for a classification that has been described for only 4 years, and should rapidly see an increase in its use due to its adoption by French vascular medicine teachers. In this context, it might be interesting to evaluate the methods of teaching a classification to vascular physicians, a specialty in its own right, recently individualized in France (18). It also has the added benefit of being a predictive marker of maximum walking distance (15). A first American consensus about Doppler waveform interpretation has just been released in July 2020 to try to clarify the way of reporting Doppler waveforms in clinical practice but it did not address the reliability of Doppler waveform analysis (19). We must also mention a risk that will have to be avoided in the future: that the Saint-Bonnet classification will not be massively adopted by vascular physicians leading to the coexistence of 3 distinct classifications and that users of the last classification will contribute to the heterogeneity of practices. The best way to avoid this risk is through continuing education but also to prove the relevance of this classification for the stratification of vascular risk or patient management.

Finally, we can hope that the use of deep learning type artificial intelligence techniques will quickly help us in the description of DWs.

## Study Limitations

The main limitation of this study is that we have selected the DW to get a representative panel, but these DW were not drawn at random. In addition, in the previous American and Chinese studies, the reliability estimates of the four-category classification were not carried out with these same DW, which complicates the comparison (9, 10). Moreover, DW were presented without medical context. This is a limitation related to the objective of establishing a classification of DW, independently of the medical context, the age of the patient and implicitly, the arterial bed examined. Another limitation of this study is the participation on a voluntary basis, potentially selecting vascular physicians already sensitized and interested in the subject. We believe that a good classification should be independent of the medical context or vascular beds and should not state whether the DW is normal or abnormal. Finally, we were not able to study intra-observer reliability or make a comparison with a five-category classification (like Descotes classification).

## Conclusion

The inter-rater reliability of 13-category Saint-Bonnet arterial Doppler classification among 20 DW and 110 vascular physician raters is weak to moderate, however unlike the other classifications, it seems to be self-sufficient, meaning that it is sufficiently precise so that the user does not need to complete its description. There is a significant heterogeneity in the use of arterial Doppler classifications in France. These results encourage an assessment of the reliability of this classification in trained physicians and coordinated teaching of the most reproducible classification.

## Data Availability Statement

The raw data supporting the conclusions of this article will be made available by the authors, without undue reservation.

## Ethics Statement

Written informed consent was obtained from the individual(s) for the publication of any potentially identifiable images or data included in this article.

## Author's Note

The authors do hereby declare that all illustrations and figures in the manuscript are entirely original and do not require reprint permission.

## Author Contributions

DL and GM: literature search, data analysis, data collection, data interpretation, writing the report, and final approval of the manuscript. JG, J-ET, CP, and LO: literature search, data analysis, data interpretation, revising the intellectual content, and final approval of the manuscript. All authors contributed to the article and approved the submitted version.

## The SFMV Pad Study Group

Detriche Gregoire, Le Hello Claire, Martin Myriam, Prigent Romain, Tadj Djamel, Allart Jean-Dominique, Bressollette Luc, Boursaly Gilles, Chleir Franck, Basthier Guillaume, Ramstein Julien, Beliard Samuel, Lestage Bruno, Jurus Christine, Nedey Charles, Claudel Guillaume, Salem Tewfik, Bozon Guillaume, Frigul Boris, Boistel Bernard, Perrinet Florence, Bertin Antoine, Weibel Shah Stephan, Dadon Michel, Tissot Anne, Roncato Christophe, Benayad Haroun, Knauer Vincent, Asius Jean-Pascal, Beaumont Myriam, Damour Jean- François, Diard Antoine, Rastel Didier, Salaun Pierre, Jeancolas Julien, Monsallier Jean Michel, Chanut Myriam, Maillard Jean-Patrick, Bettarel-Binon Catherine, Ally Benjamin, Lacroix Philippe, Toffin Luc, Ballini Odile, Ouvry Pierre, Falchero Catherine, Rouquet Bruno, Purser Christine, Leborgne Sophie, Adrien Stephane, Chamberod Remi, Fourtines Armelle, Bouchet Jacques, Le Brun Charles-Eric, Benchikh Smail, Journeau Louis, Briche Nicolas, Caudrelier Thomas, Mokaddem Wassim, Seinturier Christophe, Turenne Matthieu, Himpens François-Xavier, Bailly Philippe, Fournet Benoit, Karpoff Sandrine, Racadot Xavier, Malloizel-delaunay Julie, Giordana Pascal, Boennec Eve, Alves Manuel, Audin Jean Pierre, Croguennec Josselin, Sobiak Serge, Roux Jean-Christophe, Pichon Romana, Sevestre Marie-Antoinette, Seffert Benjamin.

## Conflict of Interest

The authors declare that the research was conducted in the absence of any commercial or financial relationships that could be construed as a potential conflict of interest.
