# Supplementary material for: Inter-rater Reliability of a 13-Category Arterial Doppler Waveform Classification and Practice of French Vascular Physicians
Source: Front Cardiovasc Med. 2021 May 25;8:640838. doi: 10.3389/fcvm.2021.640838 (PMC8185013; doi:10.3389/fcvm.2021.640838)
Supplement: Supplementary file 1 [file Presentation_1.PDF]

Evaluation of Doppler Waveform

The purpose of this questionnaire is to assess differences in terminology when describing Doppler Waveform in daily practice.

This part of the questionnaire presents 20 Doppler waveforms with a free text area allowing you to describe in a few words the appearance of the velocimetric morphotype. The flows are recorded with the usual settings (the scrolling speed is constant at 66mm/s).

1

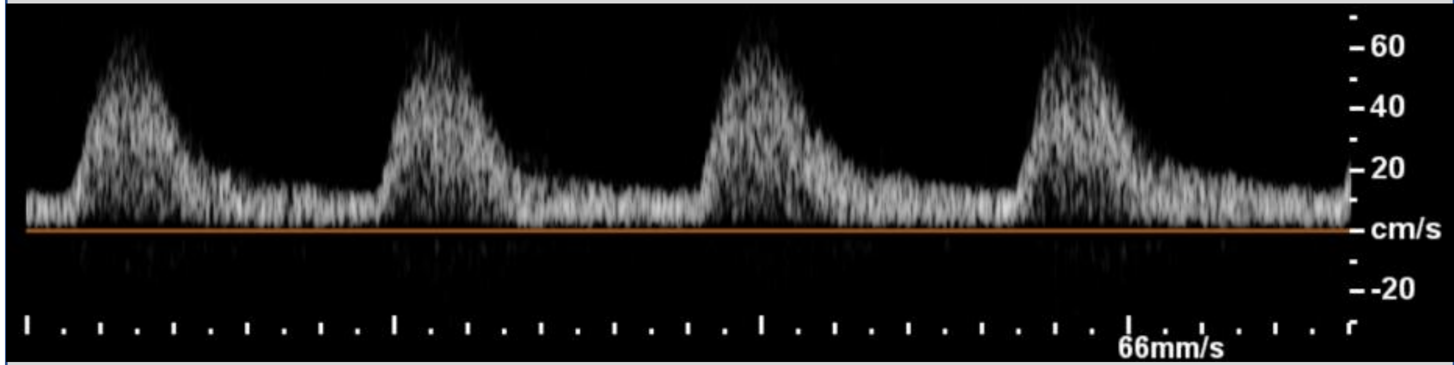

Can you describe this doppler waveform in a few words, as you might in a medical examination report?

2

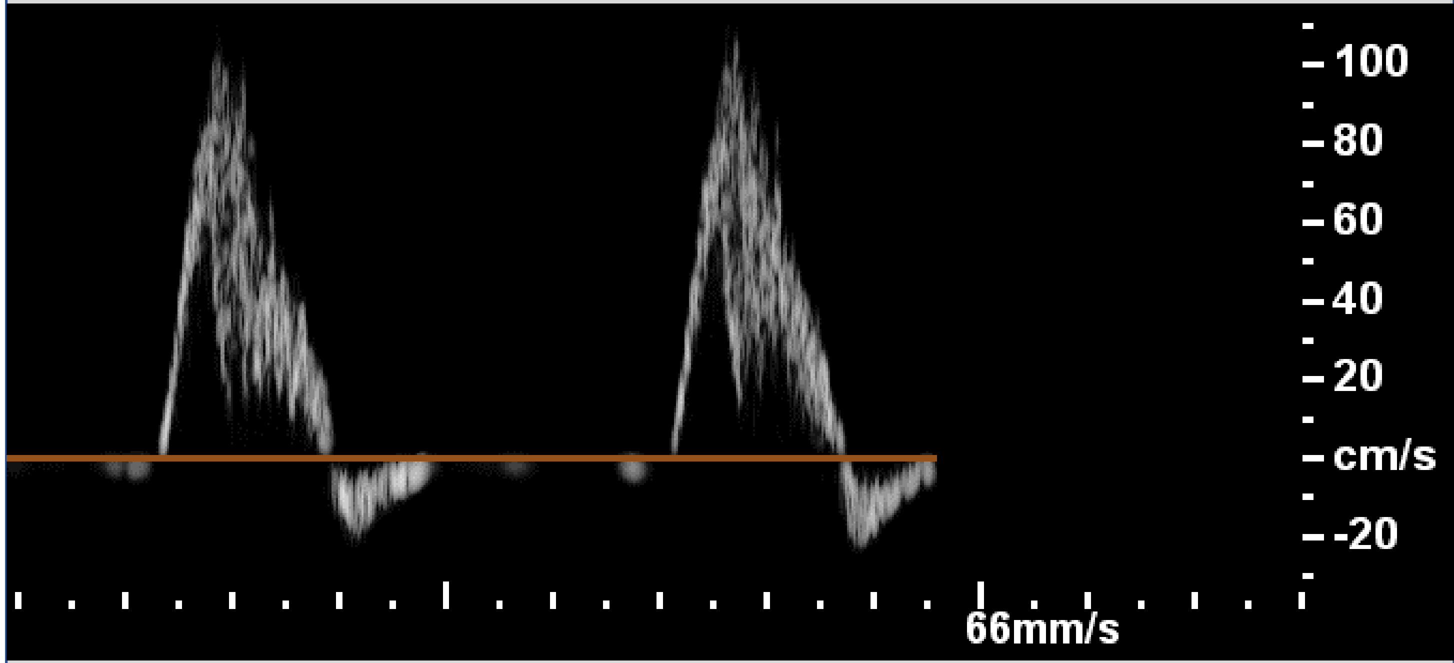

Can you describe this doppler waveform in a few words, as you might in a medical examination report?

3

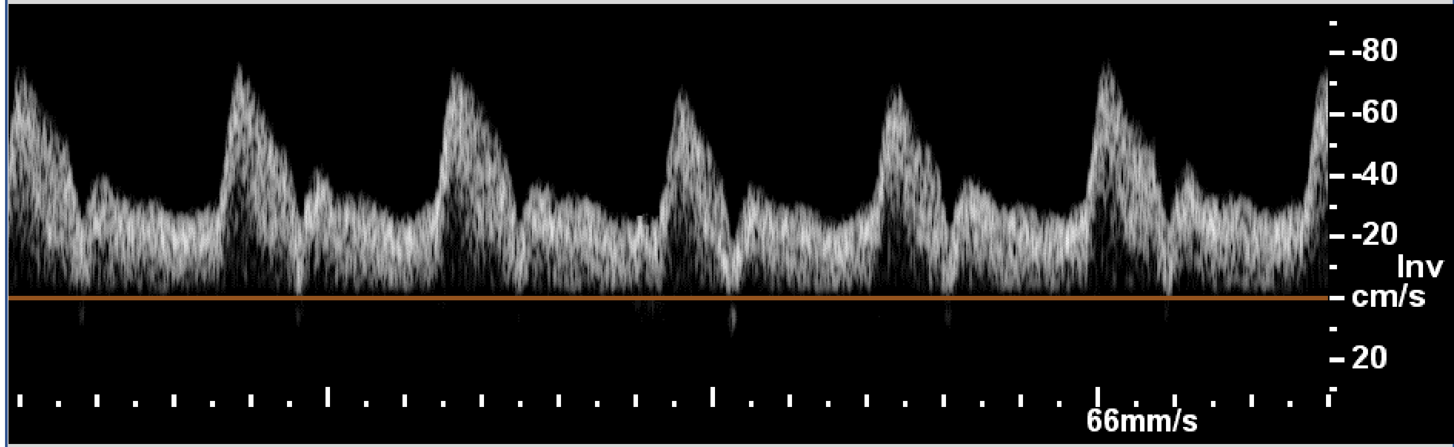

Can you describe this doppler waveform in a few words, as you might in a medical examination report?

4

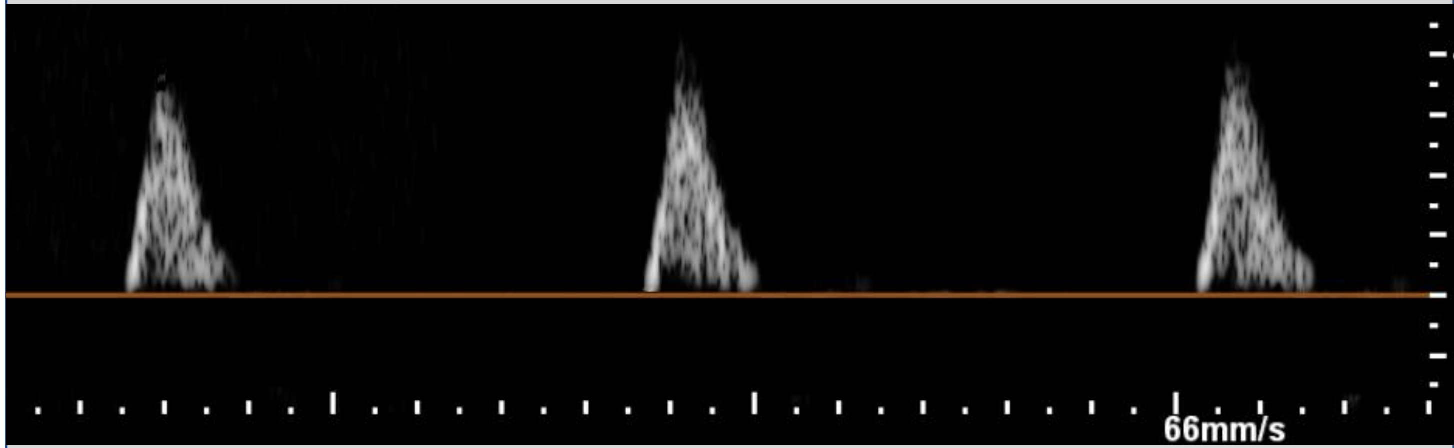

Can you describe this doppler waveform in a few words, as you might in a medical examination report?

5

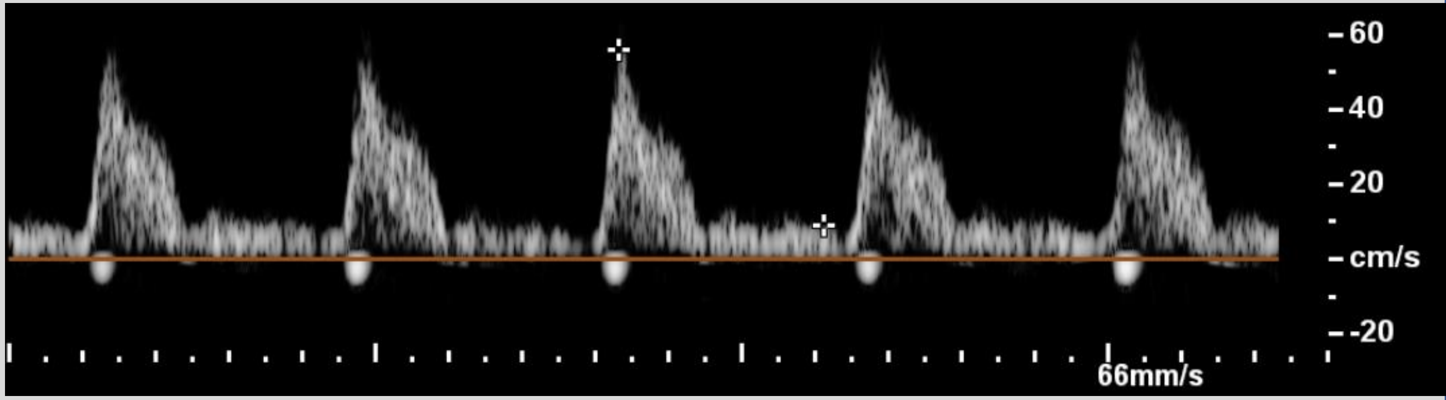

Can you describe this doppler waveform in a few words, as you might in a medical examination report?

6

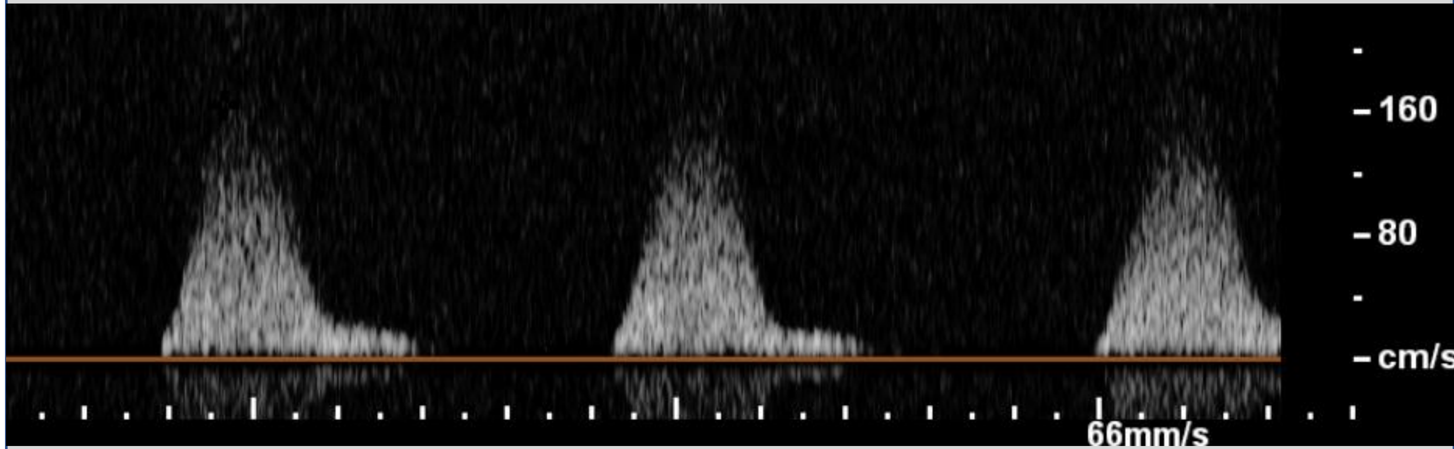

Can you describe this doppler waveform in a few words, as you might in a medical examination report?

7

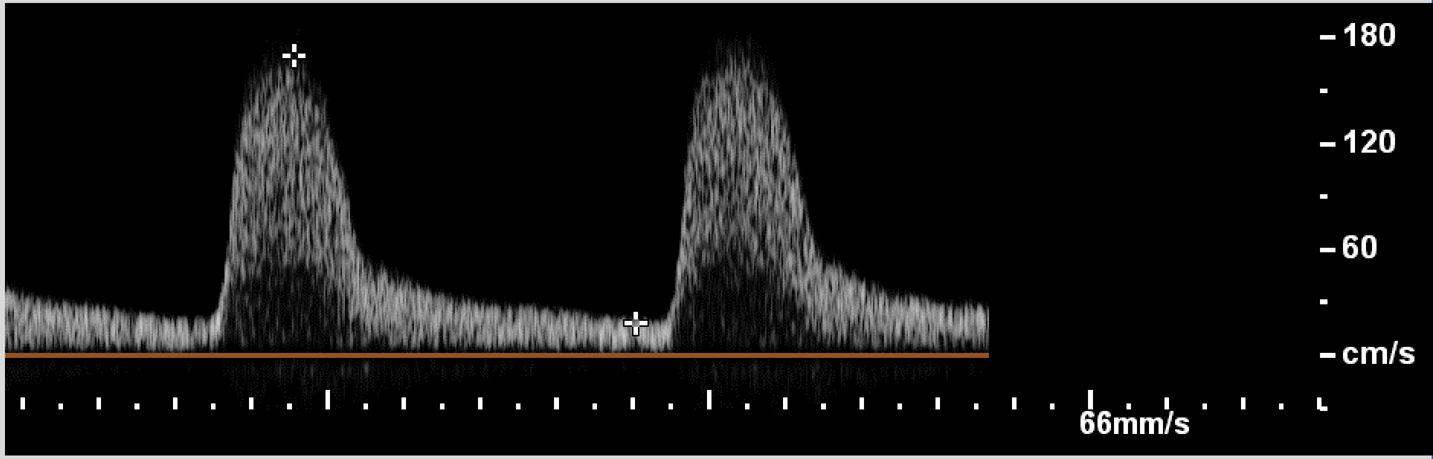

Can you describe this doppler waveform in a few words, as you might in a medical examination report?

8

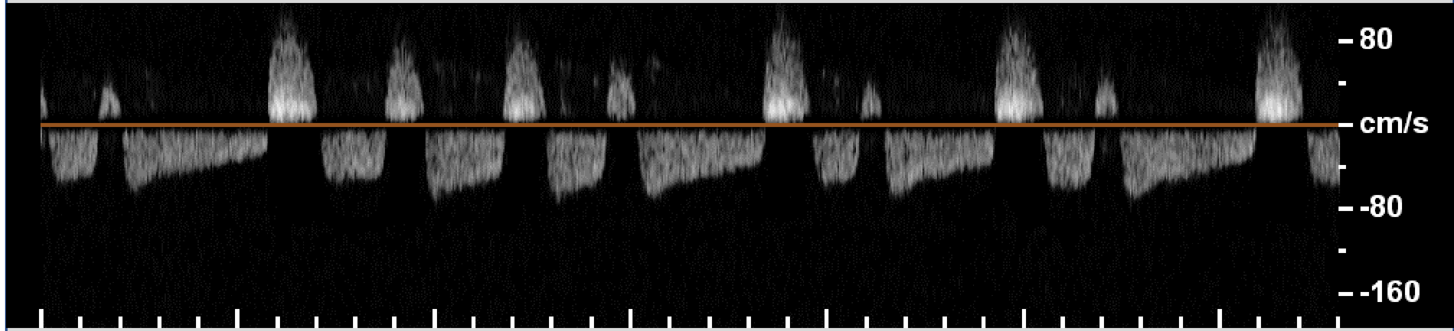

Can you describe this doppler waveform in a few words, as you might in a medical examination report?

9

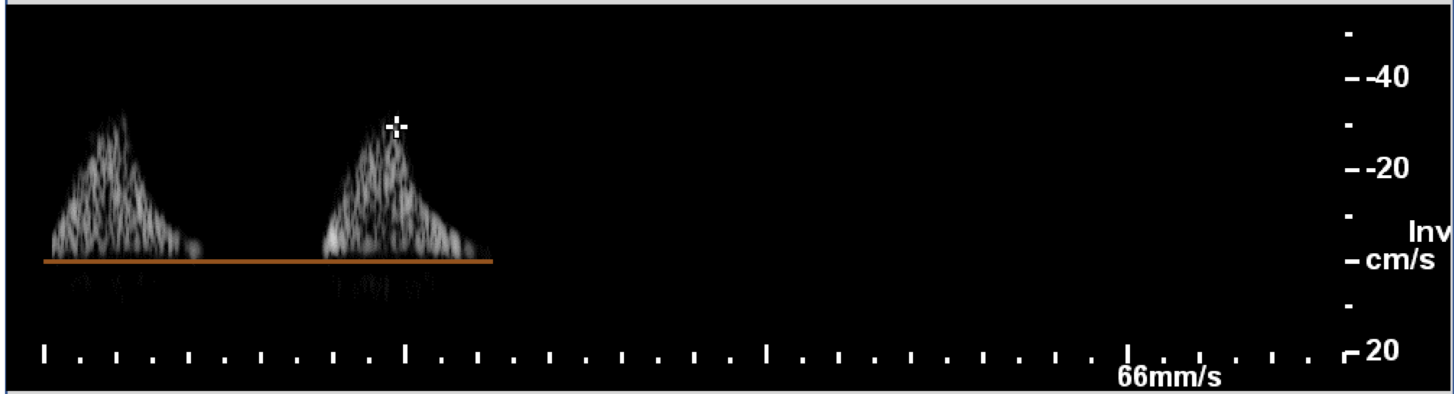

Can you describe this doppler waveform in a few words, as you might in a medical examination report?

10

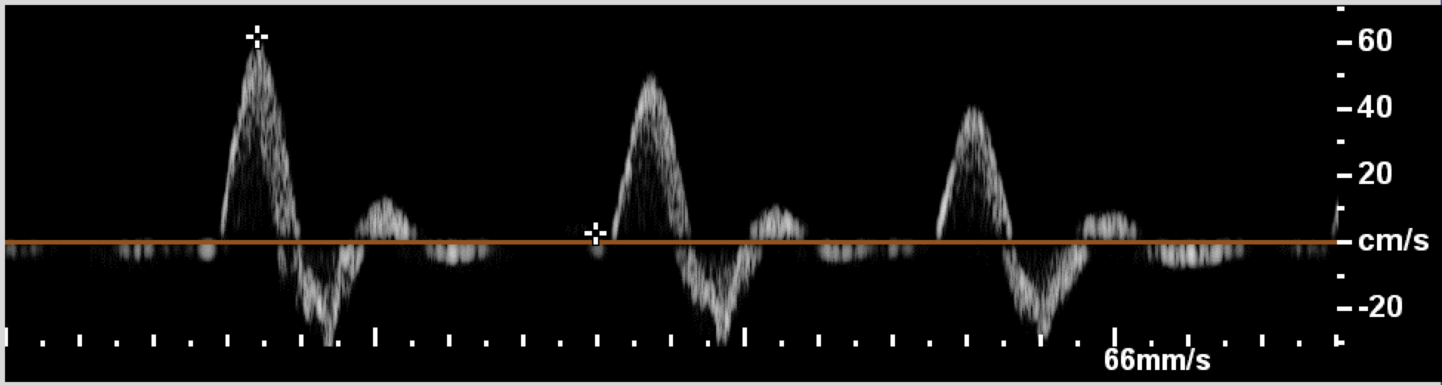

Can you describe this doppler waveform in a few words, as you might in a medical examination report?

11

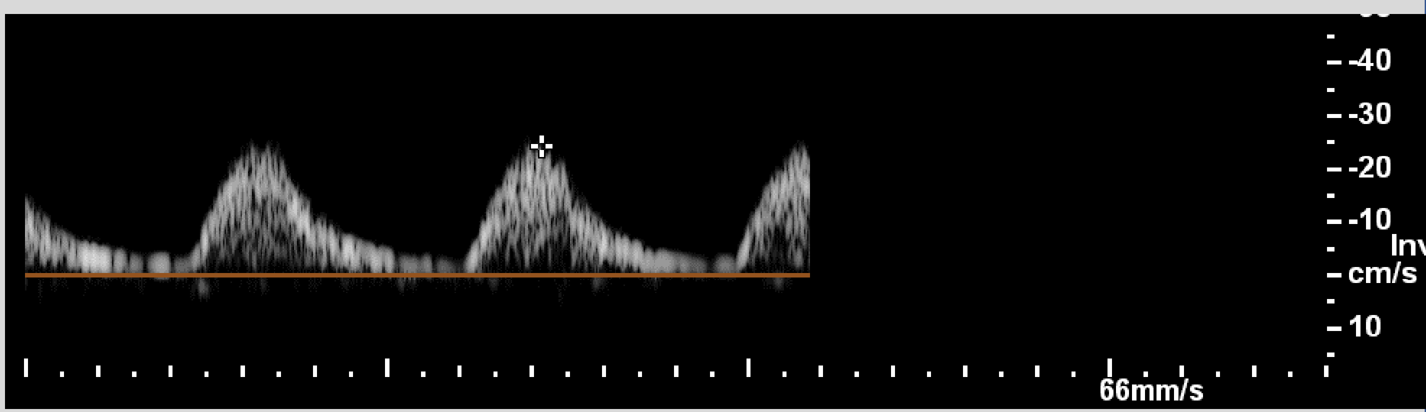

Can you describe this doppler waveform in a few words, as you might in a medical examination report?

12

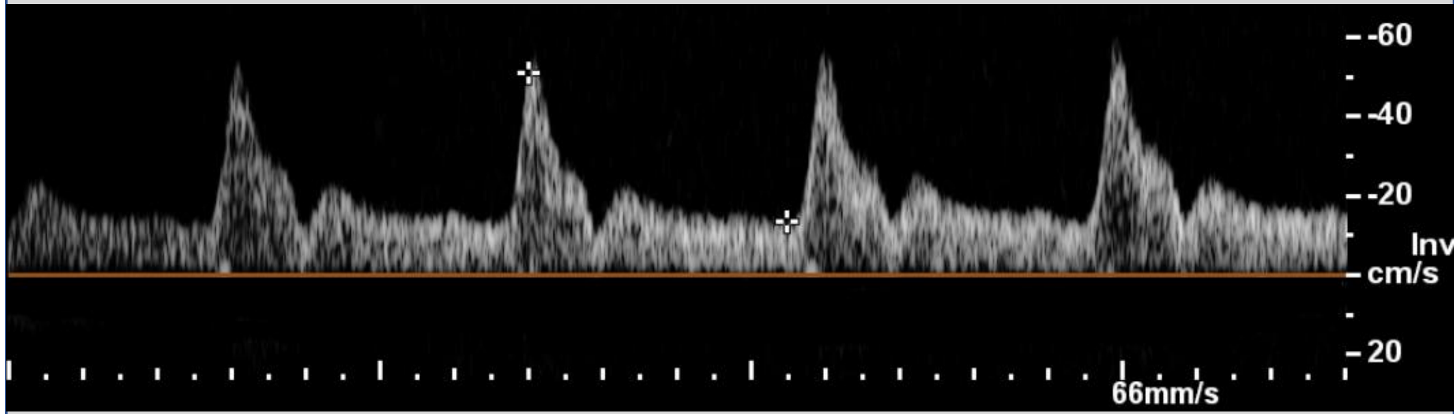

Can you describe this doppler waveform in a few words, as you might in a medical examination report?

13

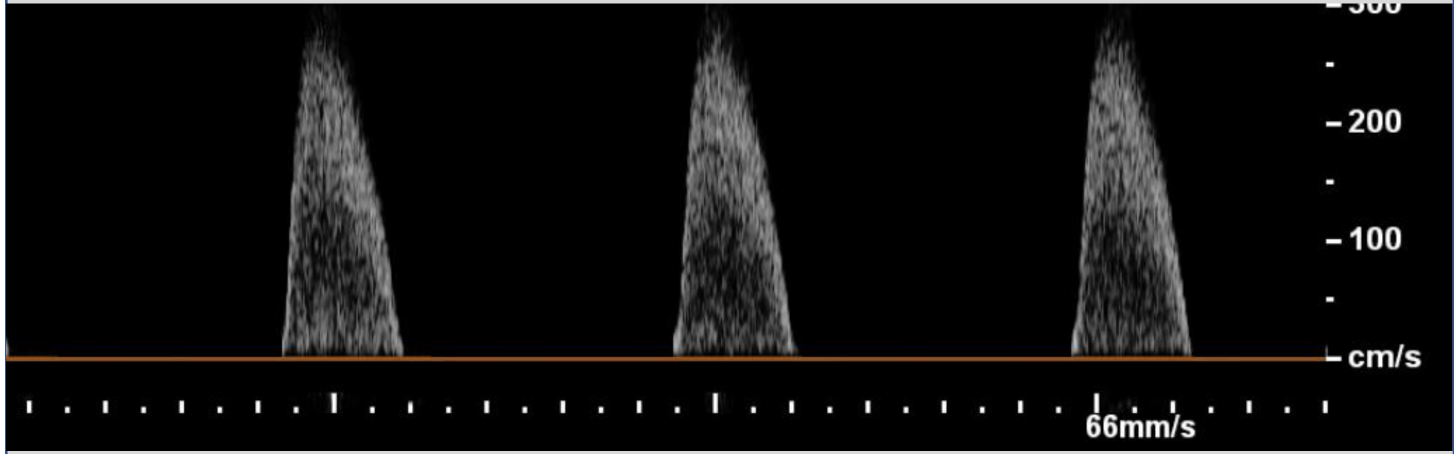

Can you describe this doppler waveform in a few words, as you might in a medical examination report?

14

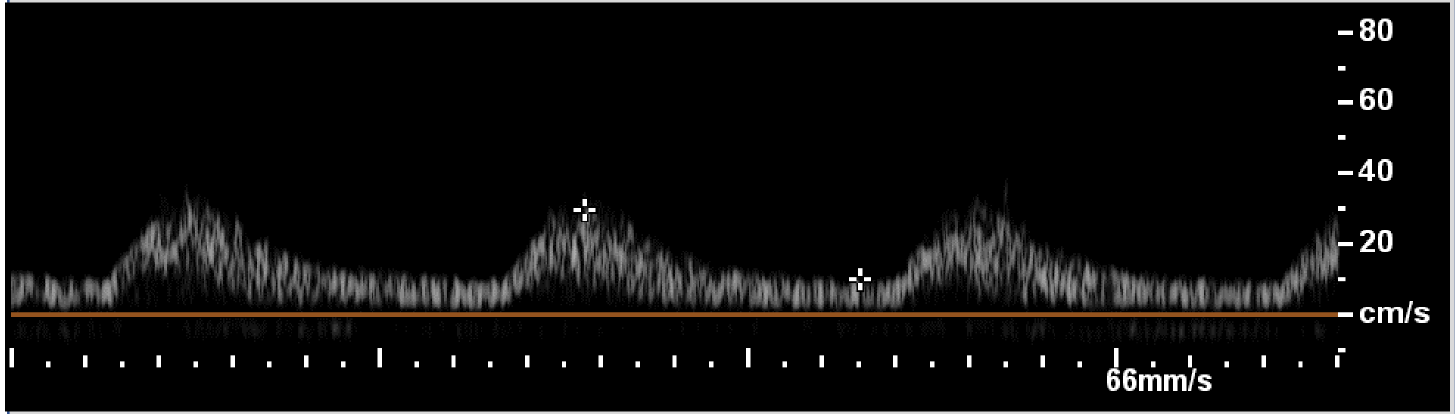

Can you describe this doppler waveform in a few words, as you might in a medical examination report?

15

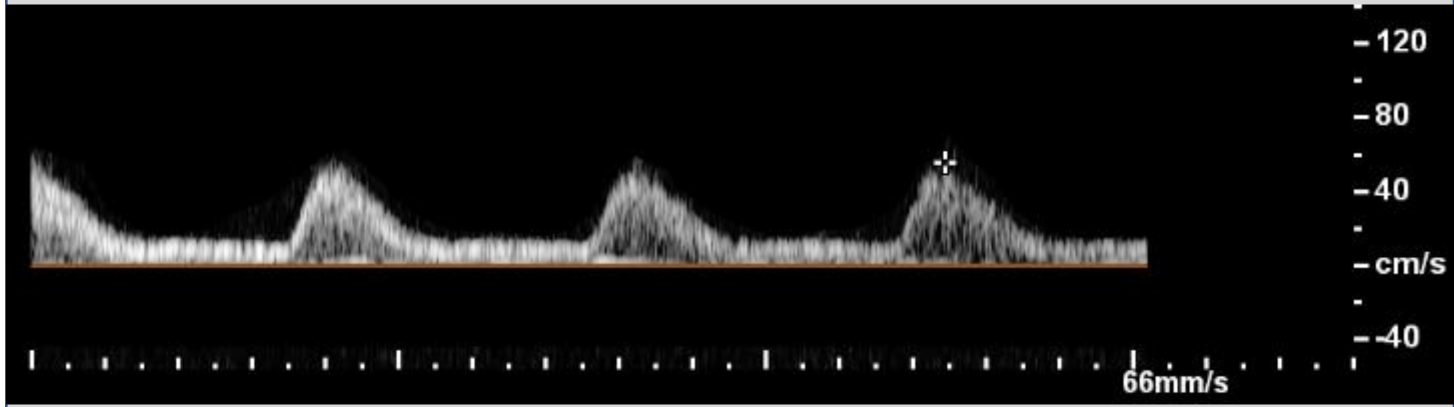

Can you describe this doppler waveform in a few words, as you might in a medical examination report?

16

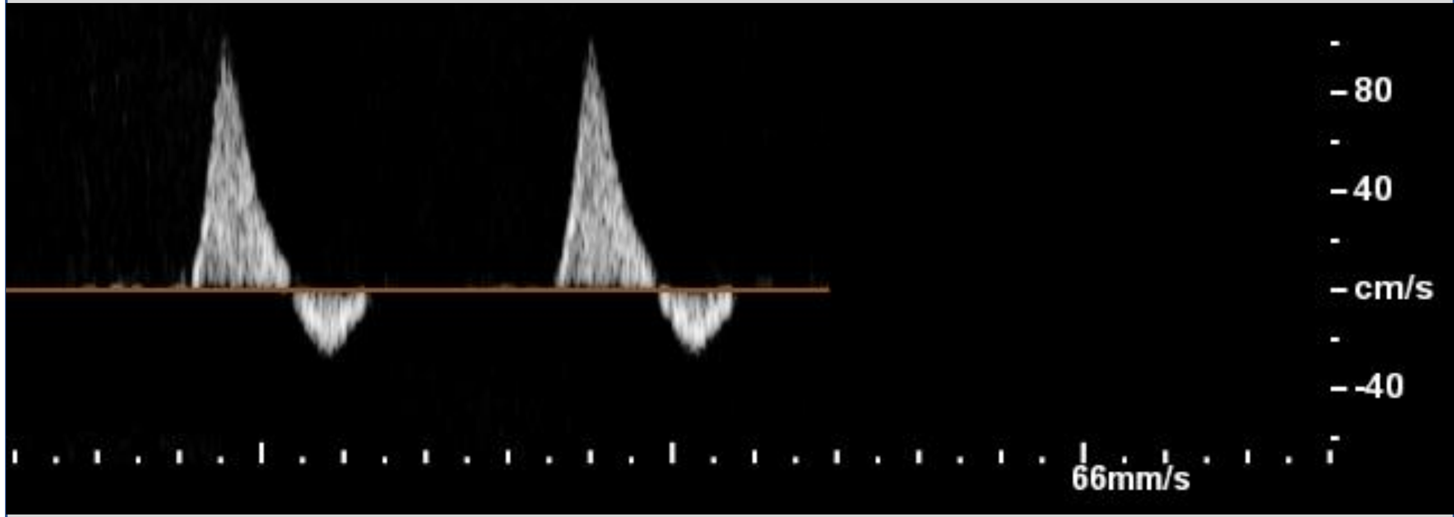

Can you describe this doppler waveform in a few words, as you might in a medical examination report?

17

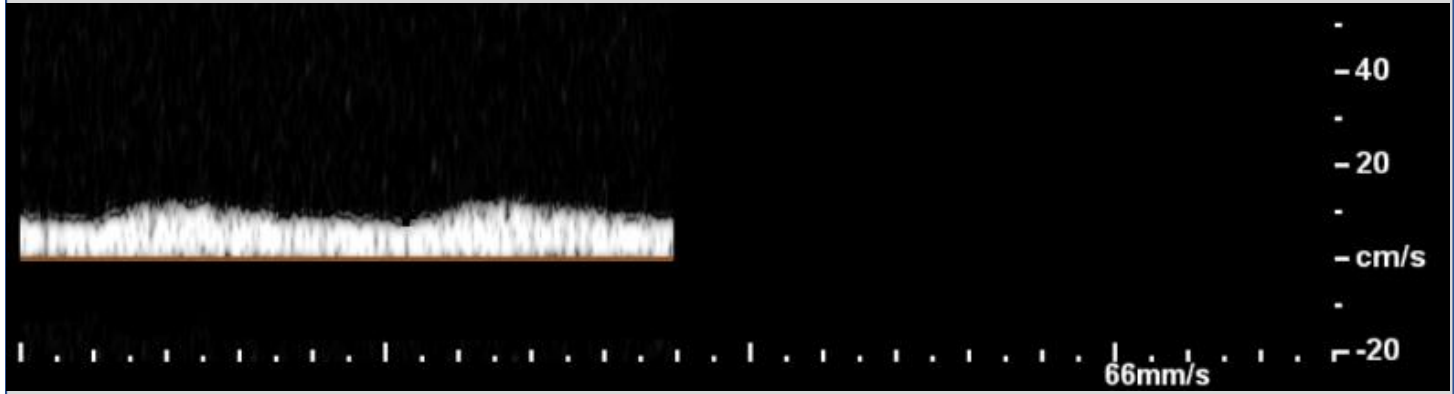

Can you describe this doppler waveform in a few words, as you might in a medical examination report?

18

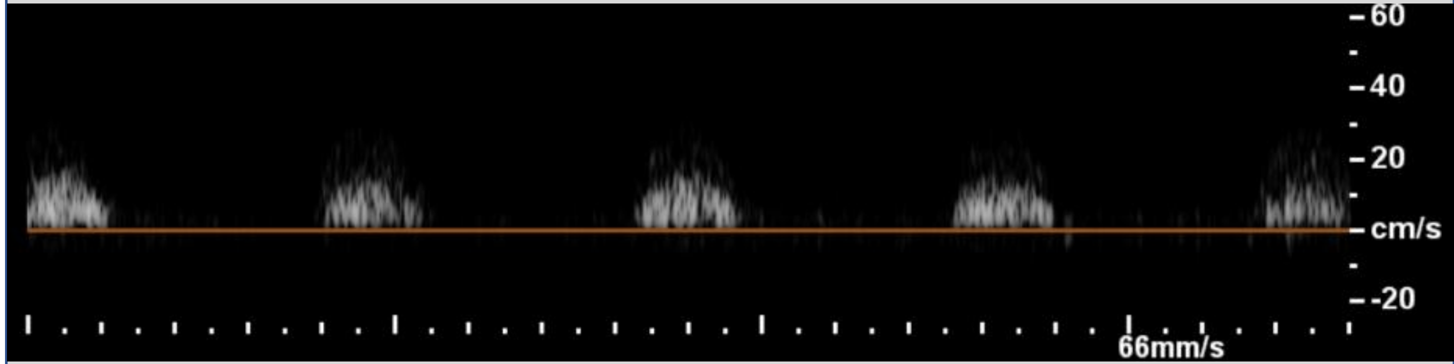

Can you describe this doppler waveform in a few words, as you might in a medical examination report?

19

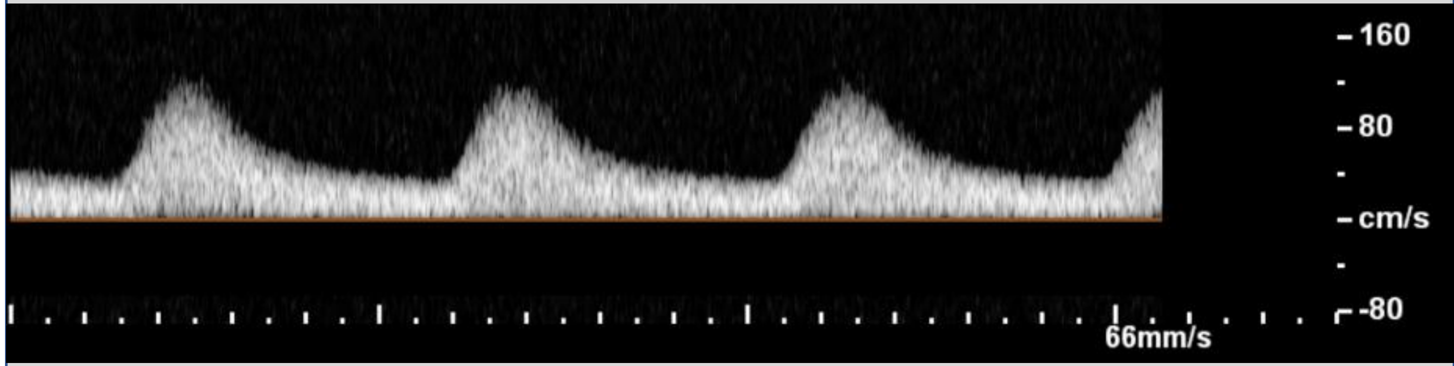

Can you describe this doppler waveform in a few words, as you might in a medical examination report?

20

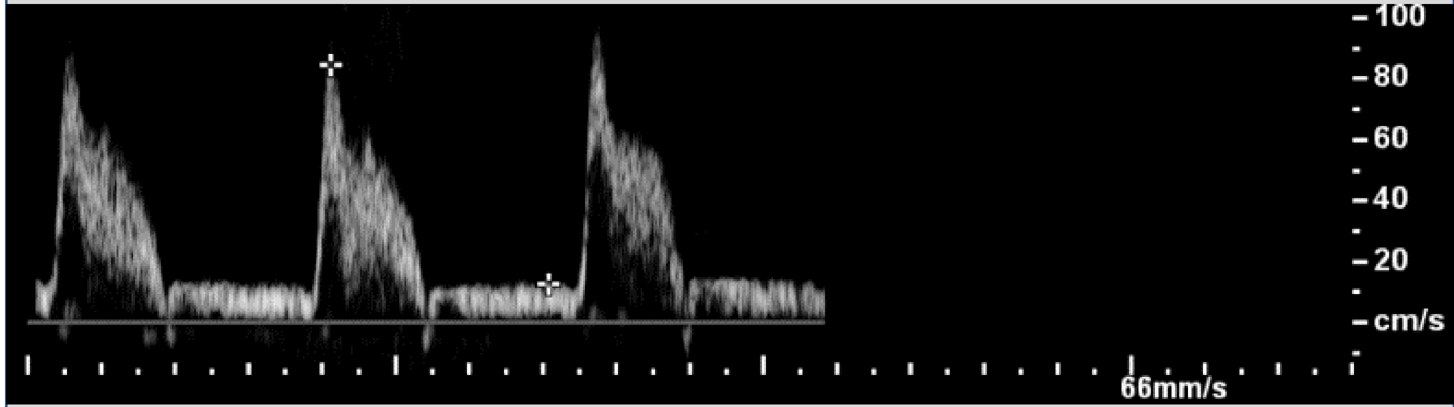

Can you describe this doppler waveform in a few words, as you might in a medical examination report?

The last part of this questionnaire asks you for the same description, this time with the help of a classification present on the left side of the screen (Saint Bonnet).

Classification of Saint Bonnet without continuous flow

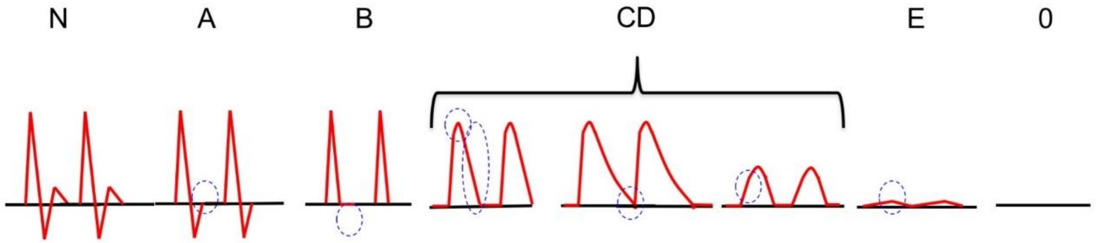

Classification of Saint Bonnet with continuous flow

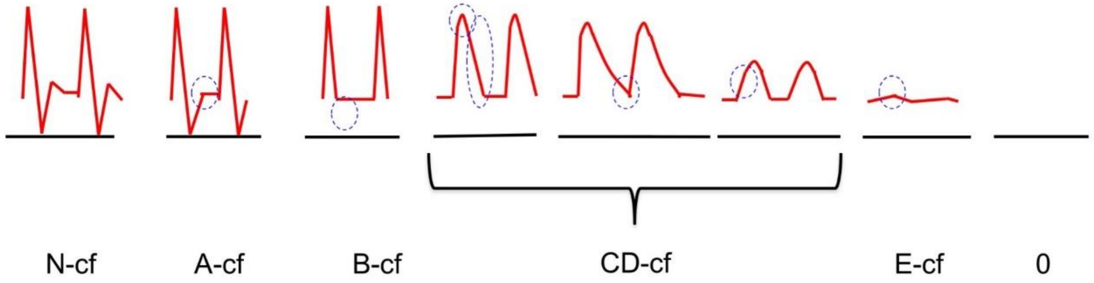

Classification of Saint Bonnet for false aneurysm flow

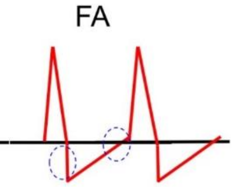

In some cases, the flow may not correspond to any flow in the classification, in which case the letter U should be used.

1

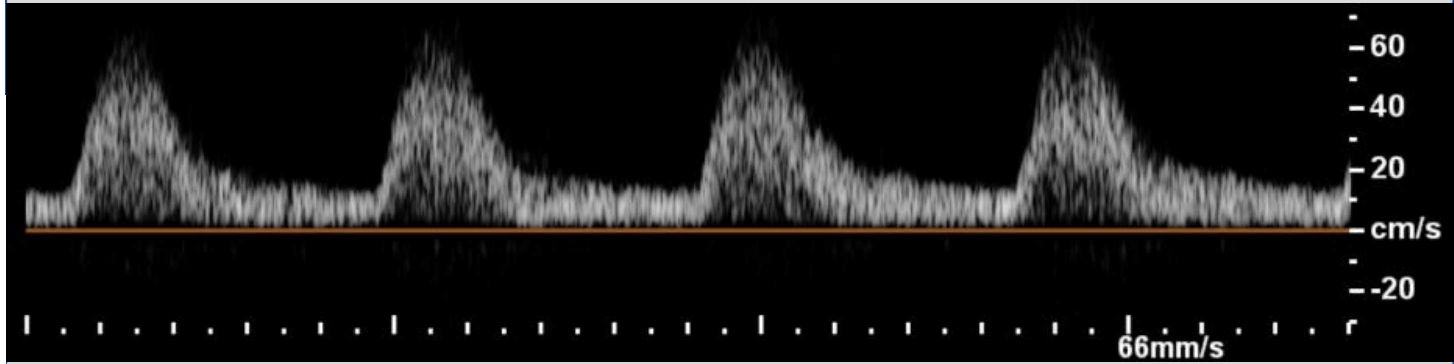

Can you describe this doppler waveform using the classification on the left side of the screen (Saint Bonnet).

Classification of Saint Bonnet without continuous flow

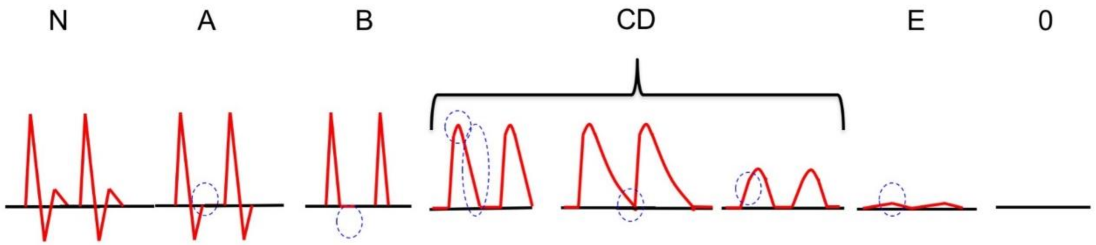

Classification of Saint Bonnet with continuous flow

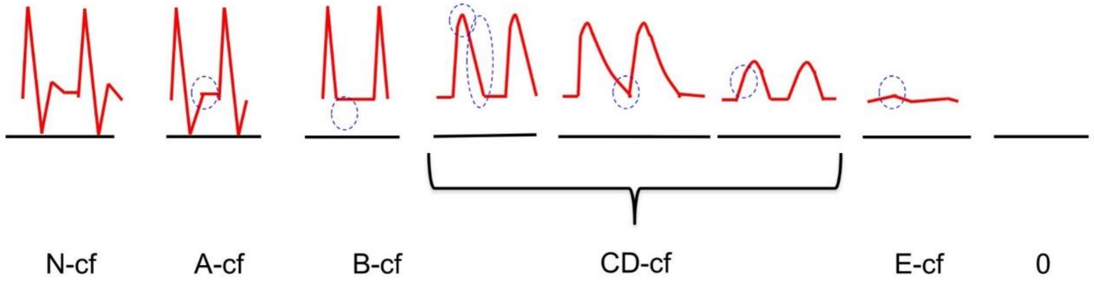

Classification of Saint Bonnet for false aneurysm flow

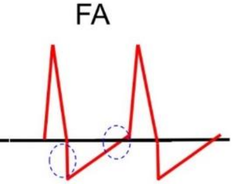

In some cases, the flow may not correspond to any flow in the classification, in which case the letter U should be used.

2

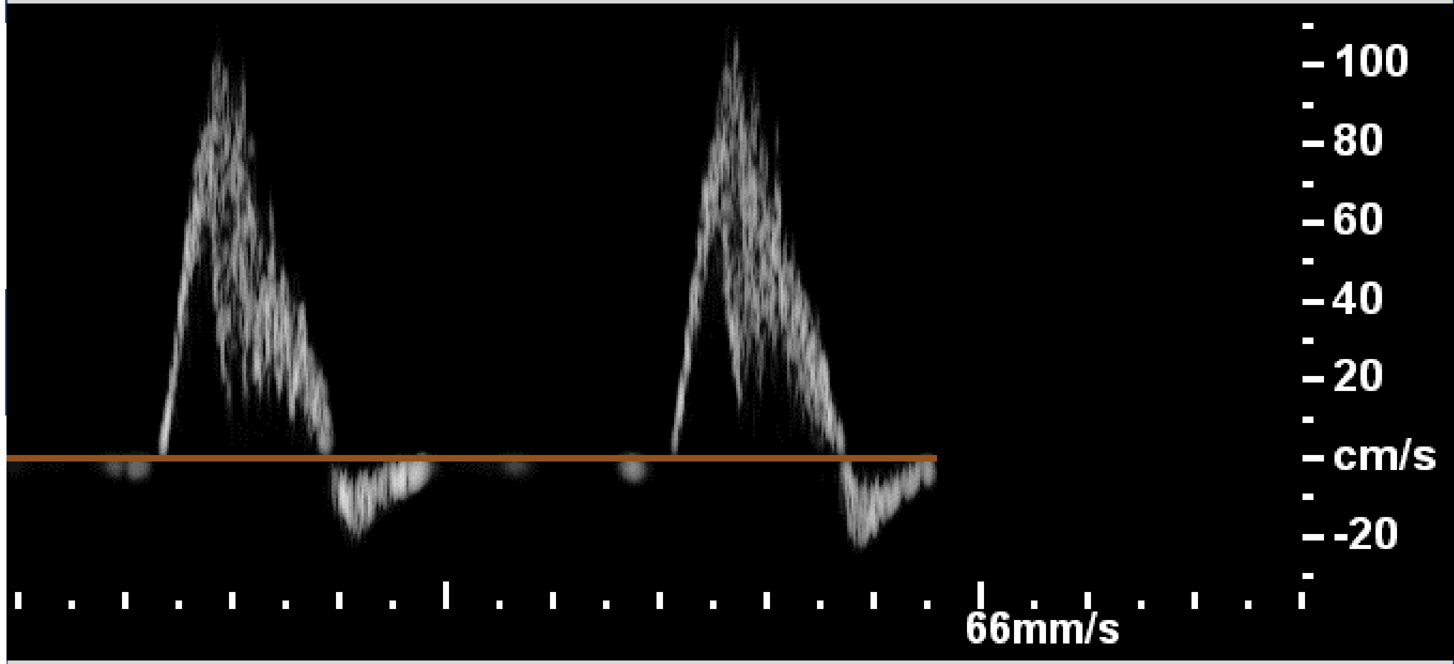

Can you describe this doppler waveform using the classification on the left side of the screen (Saint Bonnet).

Classification of Saint Bonnet without continuous flow

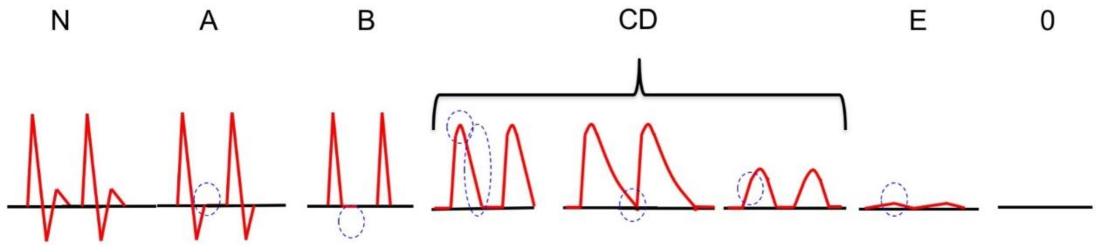

Classification of Saint Bonnet with continuous flow

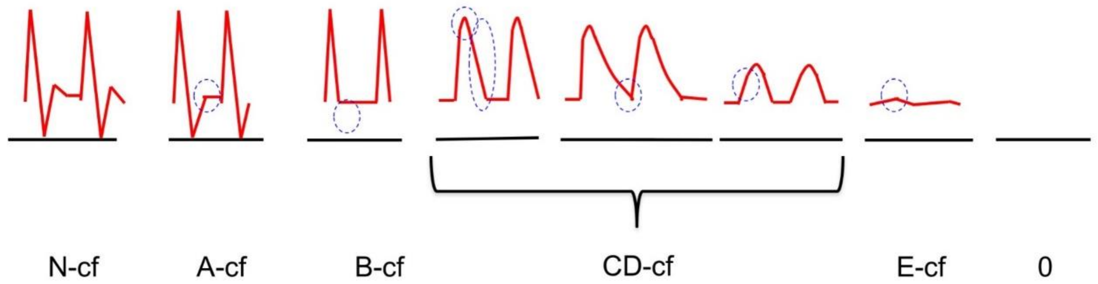

Classification of Saint Bonnet for false aneurysm flow

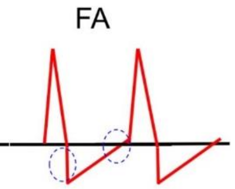

In some cases, the flow may not correspond to any flow in the classification, in which case the letter U should be used.

3

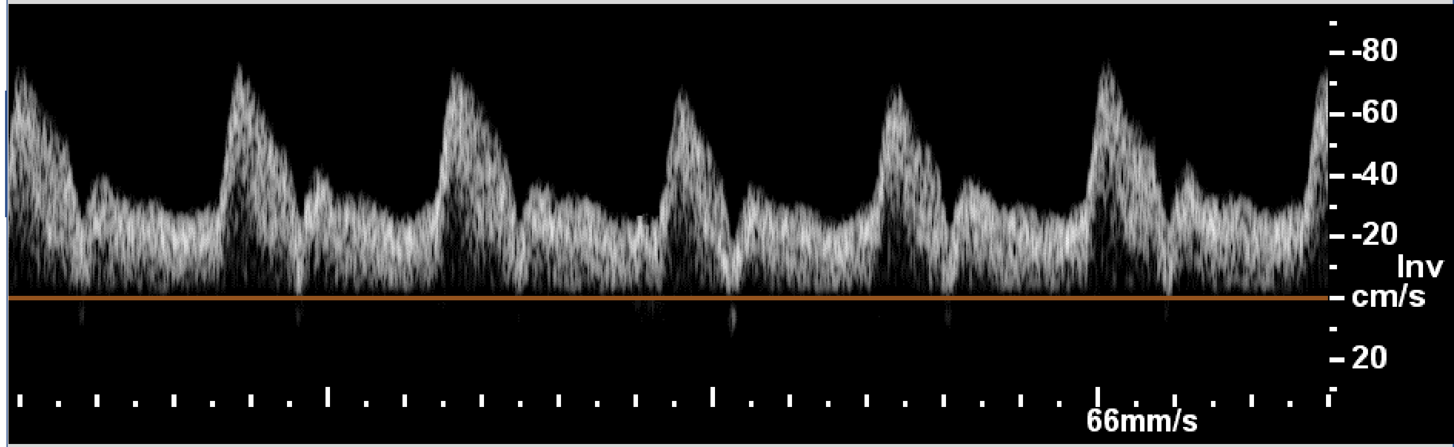

Can you describe this doppler waveform using the classification on the left side of the screen (Saint Bonnet).

Classification of Saint Bonnet without continuous flow

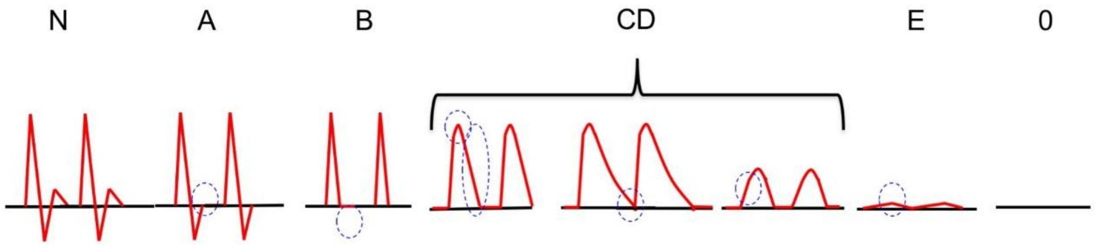

Classification of Saint Bonnet with continuous flow

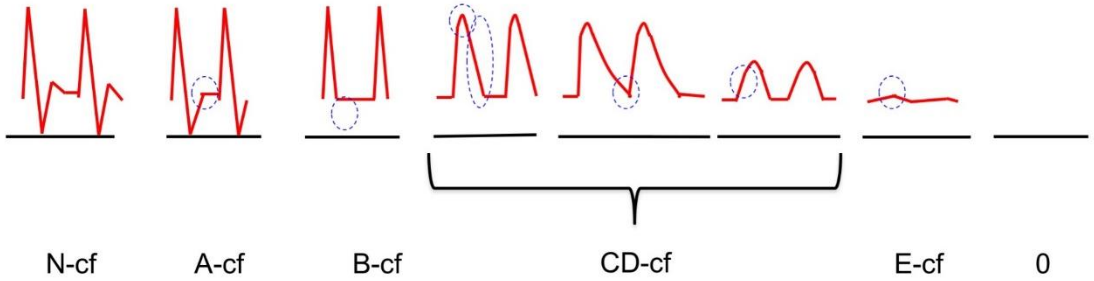

Classification of Saint Bonnet for false aneurysm flow

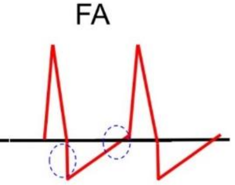

In some cases, the flow may not correspond to any flow in the classification, in which case the letter U should be used.

4

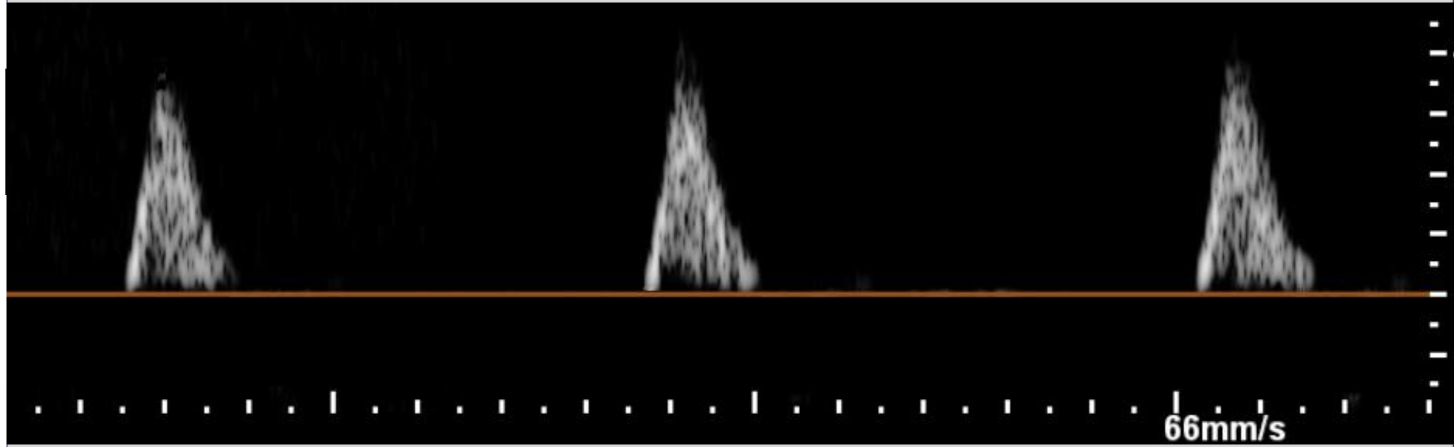

Can you describe this doppler waveform using the classification on the left side of the screen (Saint Bonnet).

Classification of Saint Bonnet without continuous flow

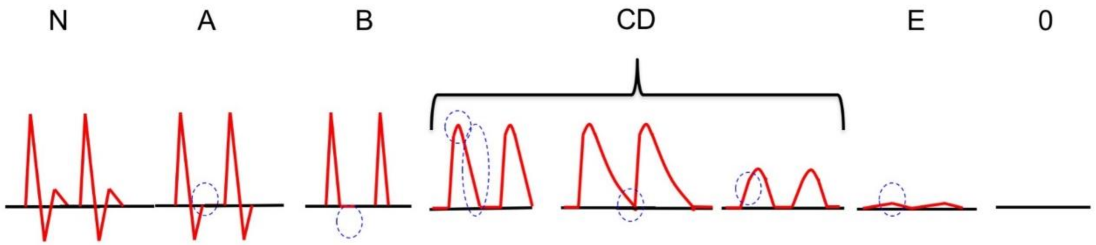

Classification of Saint Bonnet with continuous flow

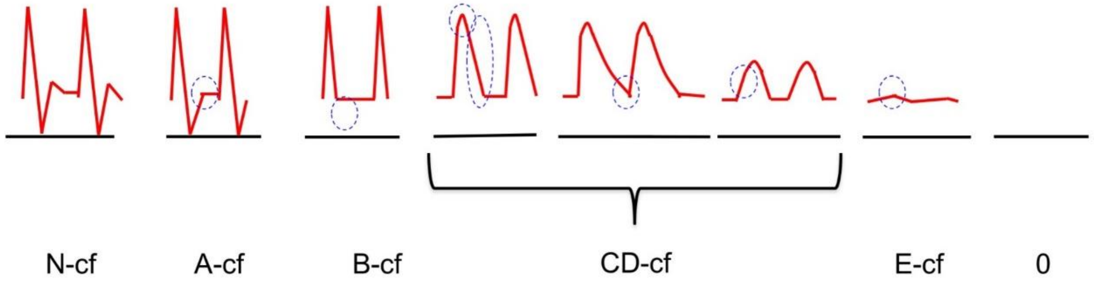

Classification of Saint Bonnet for false aneurysm flow

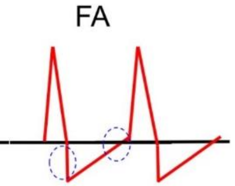

In some cases, the flow may not correspond to any flow in the classification, in which case the letter U should be used.

5

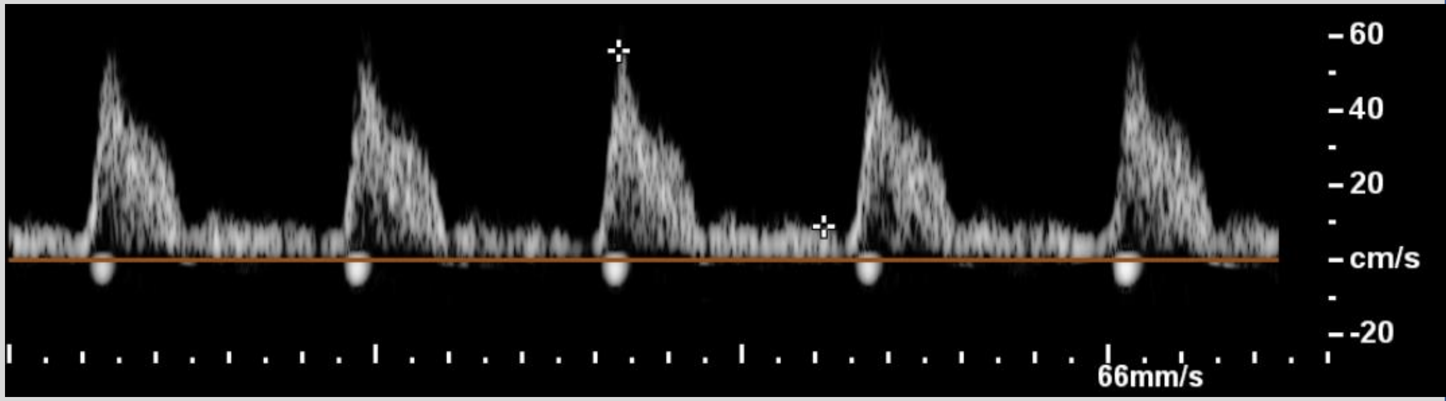

Can you describe this doppler waveform using the classification on the left side of the screen (Saint Bonnet).

Classification of Saint Bonnet without continuous flow

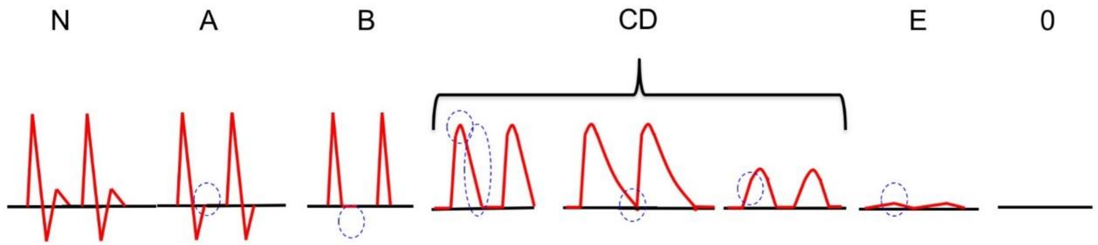

Classification of Saint Bonnet with continuous flow

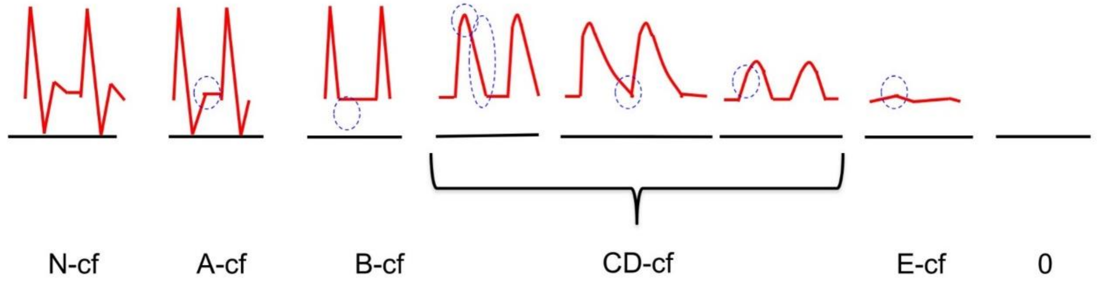

Classification of Saint Bonnet for false aneurysm flow

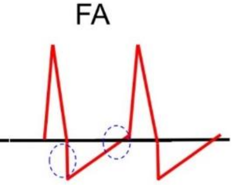

In some cases, the flow may not correspond to any flow in the classification, in which case the letter U should be used.

6

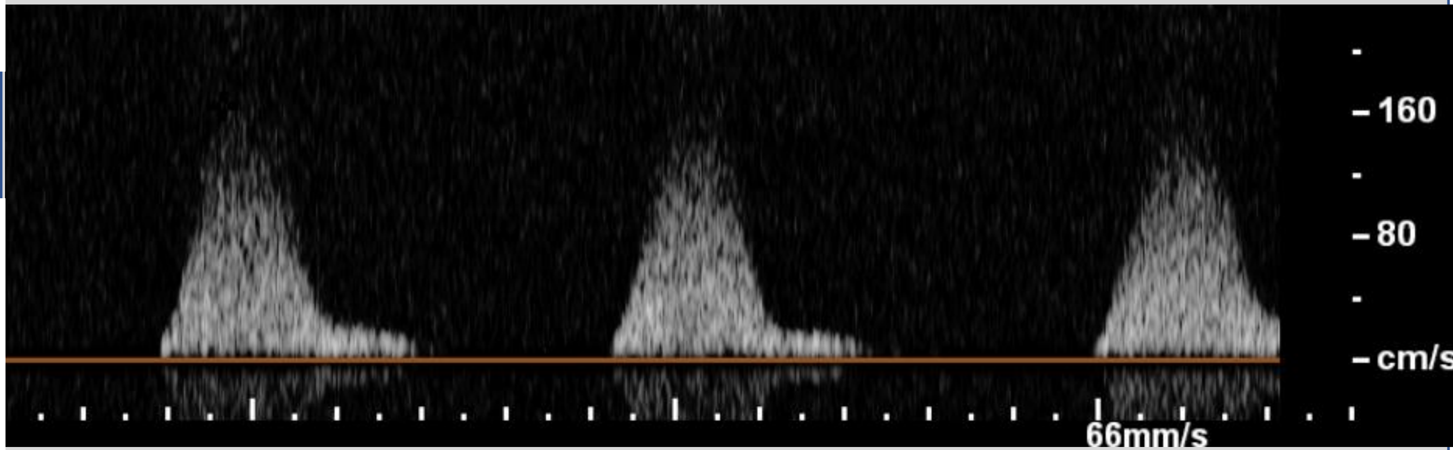

Can you describe this doppler waveform using the classification on the left side of the screen (Saint Bonnet).

Classification of Saint Bonnet without continuous flow

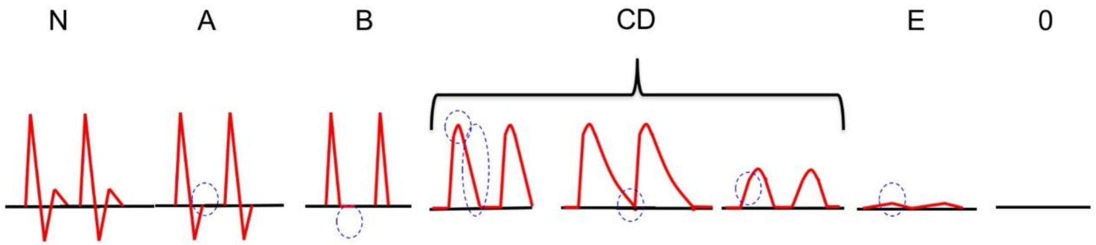

Classification of Saint Bonnet with continuous flow

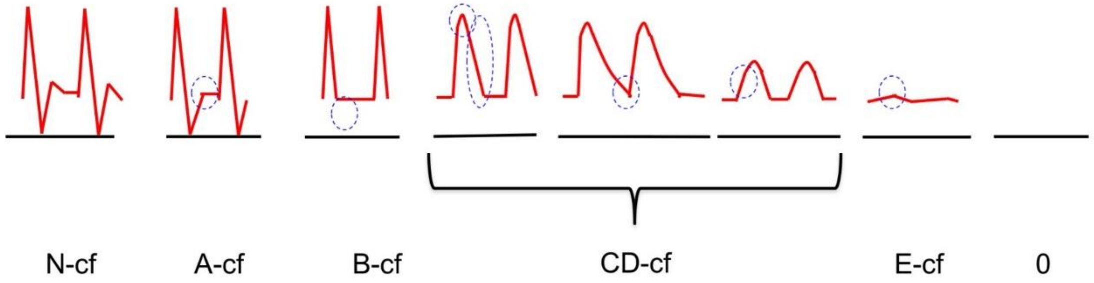

Classification of Saint Bonnet for false aneurysm flow

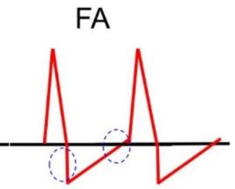

In some cases, the flow may not correspond to any flow in the classification, in which case the letter U should be used.

7

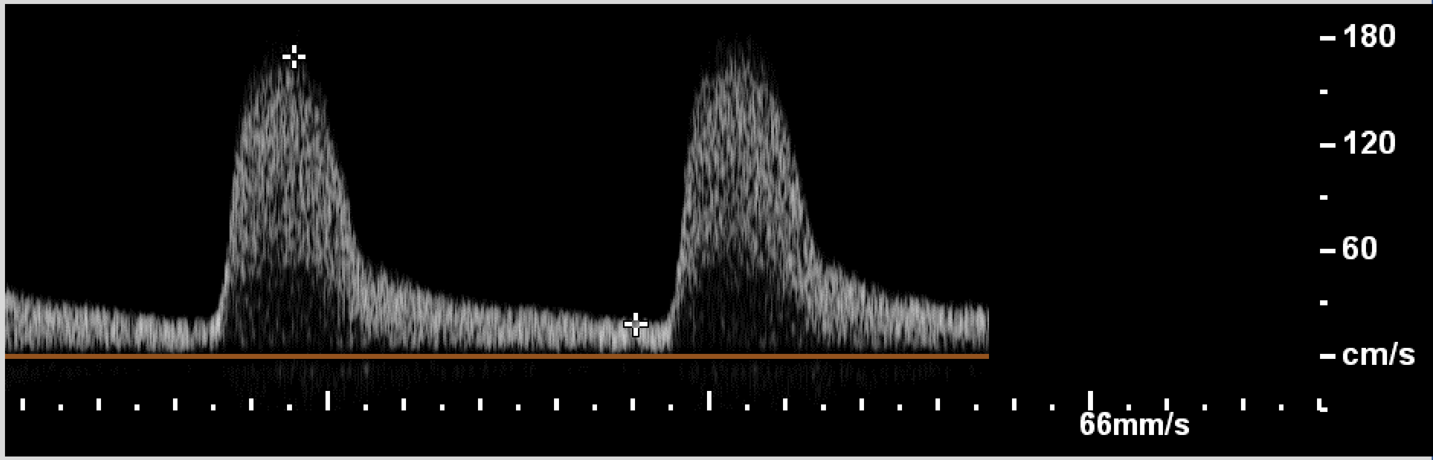

Can you describe this doppler waveform using the classification on the left side of the screen (Saint Bonnet).

Classification of Saint Bonnet without continuous flow

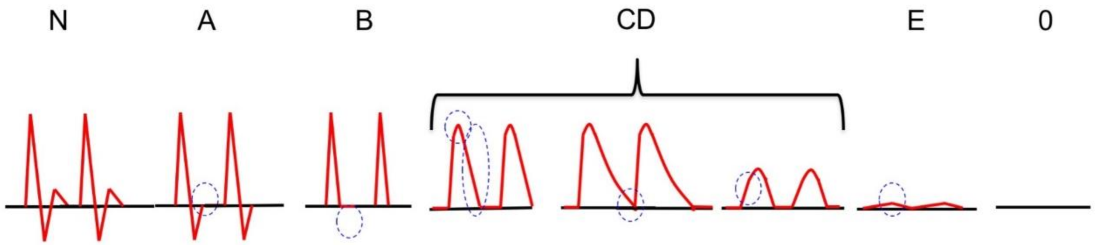

Classification of Saint Bonnet with continuous flow

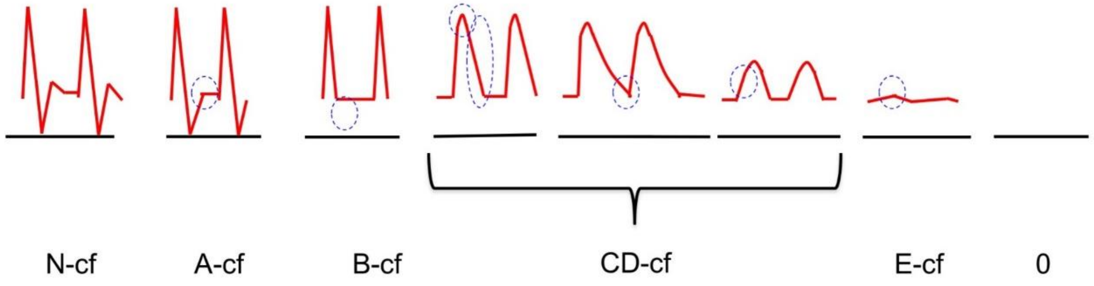

Classification of Saint Bonnet for false aneurysm flow

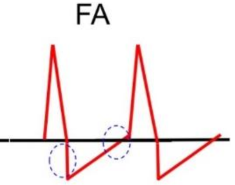

In some cases, the flow may not correspond to any flow in the classification, in which case the letter U should be used.

8

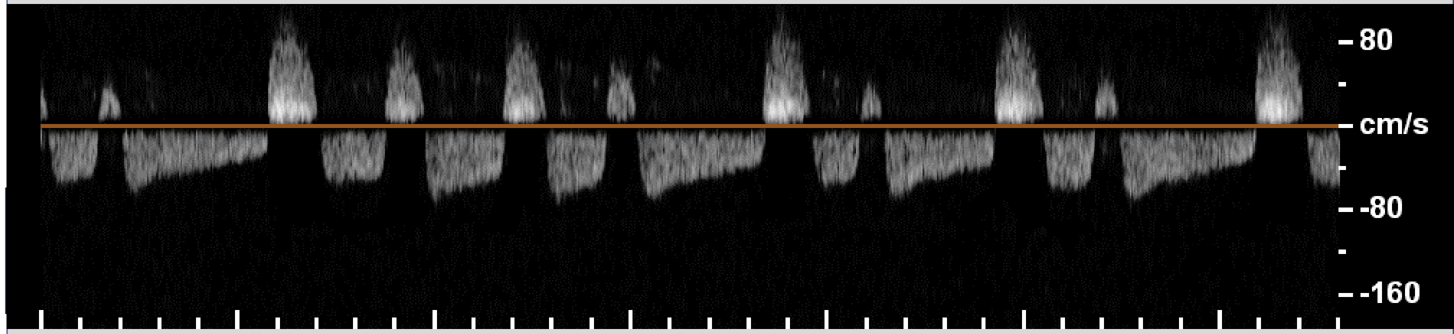

Can you describe this doppler waveform using the classification on the left side of the screen (Saint Bonnet).

Classification of Saint Bonnet without continuous flow

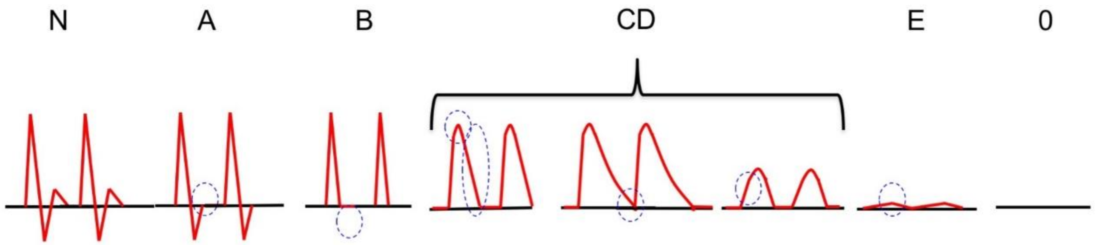

Classification of Saint Bonnet with continuous flow

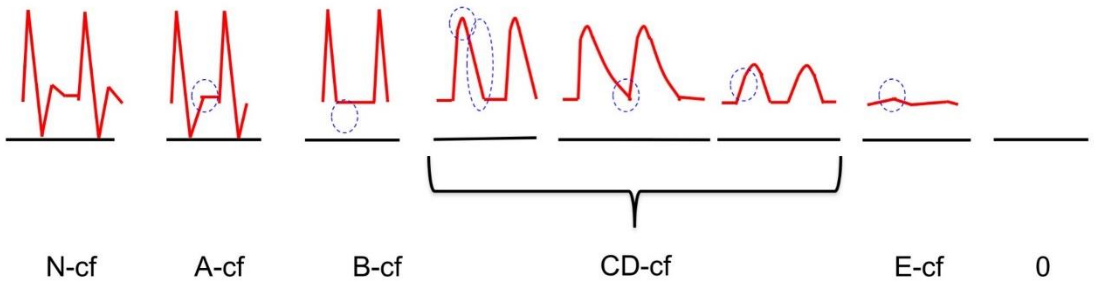

Classification of Saint Bonnet for false aneurysm flow

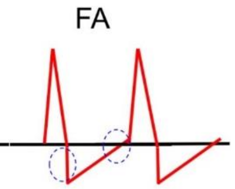

In some cases, the flow may not correspond to any flow in the classification, in which case the letter U should be used.

9

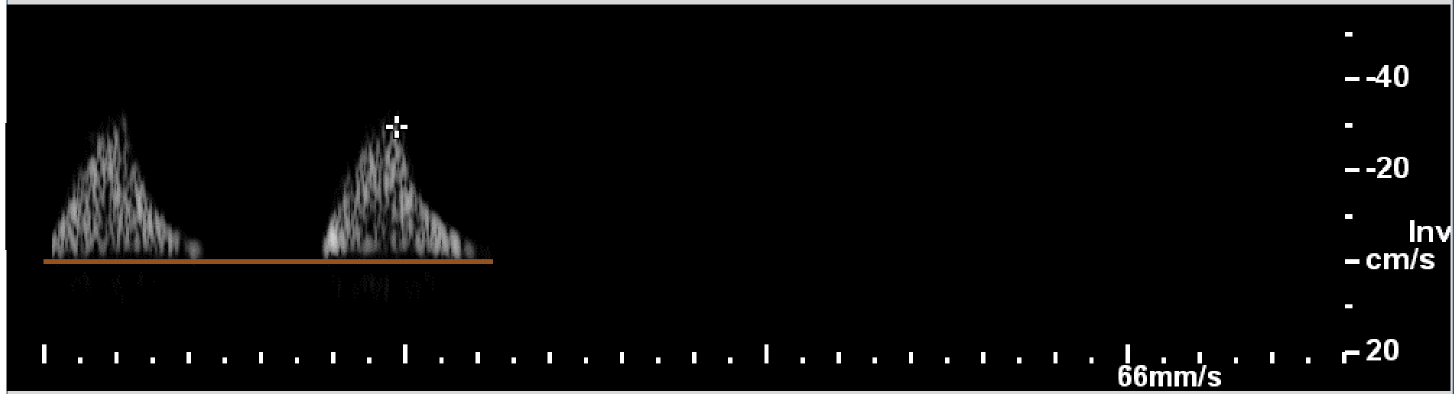

Can you describe this doppler waveform using the classification on the left side of the screen (Saint Bonnet).

Classification of Saint Bonnet without continuous flow

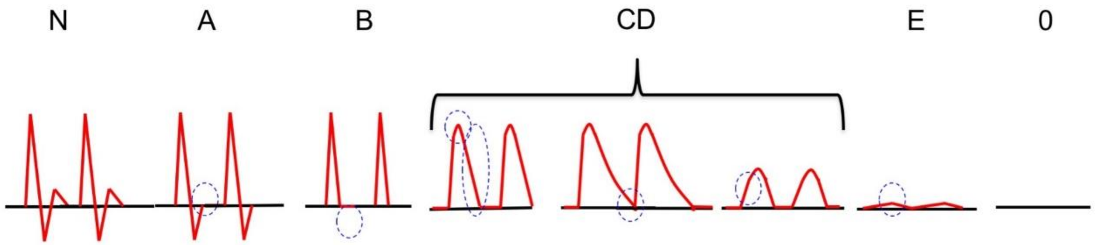

Classification of Saint Bonnet with continuous flow

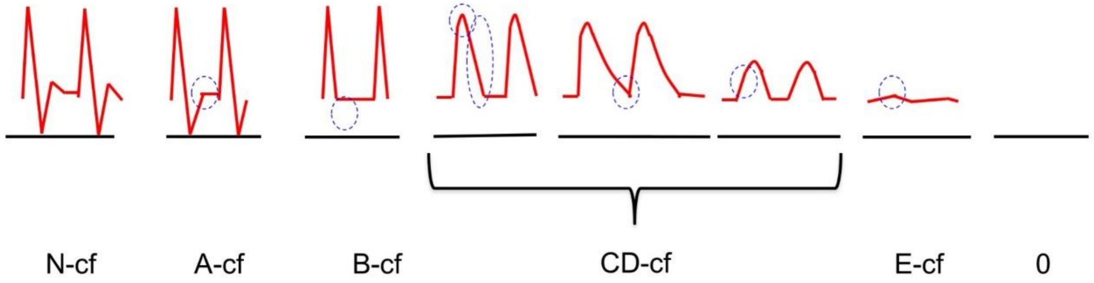

Classification of Saint Bonnet for false aneurysm flow

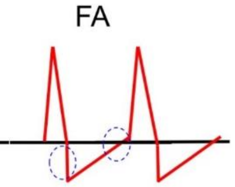

In some cases, the flow may not correspond to any flow in the classification, in which case the letter U should be used.

10

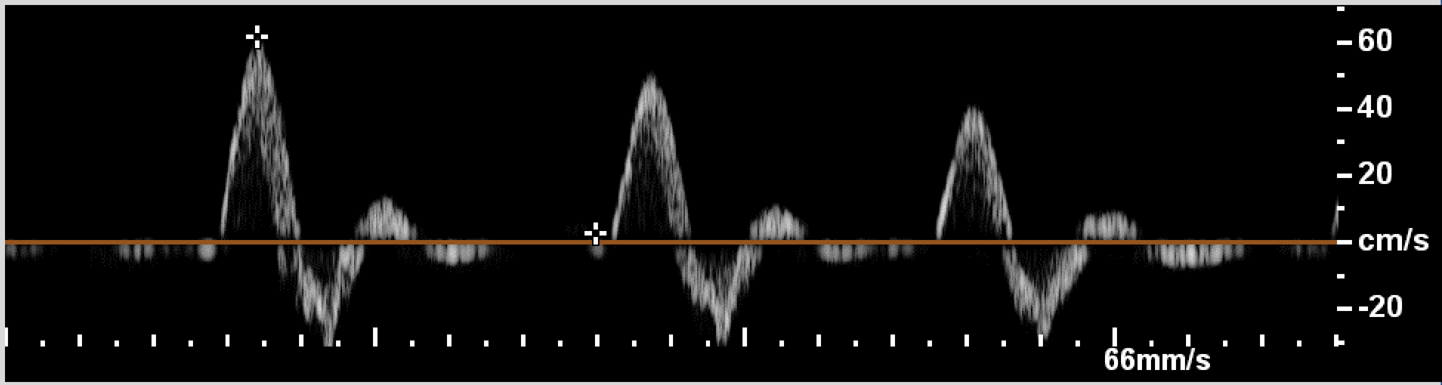

Can you describe this doppler waveform using the classification on the left side of the screen (Saint Bonnet).

Classification of Saint Bonnet without continuous flow

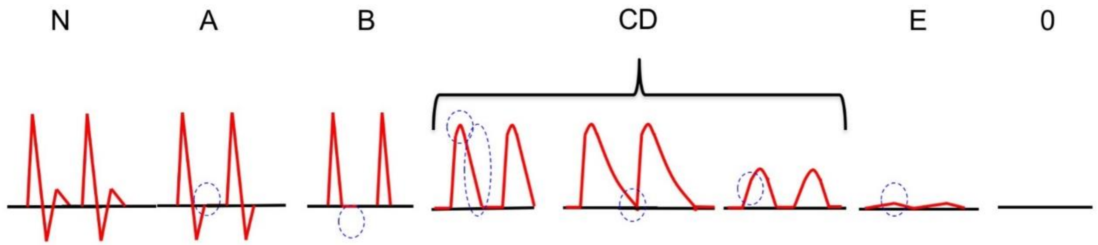

Classification of Saint Bonnet with continuous flow

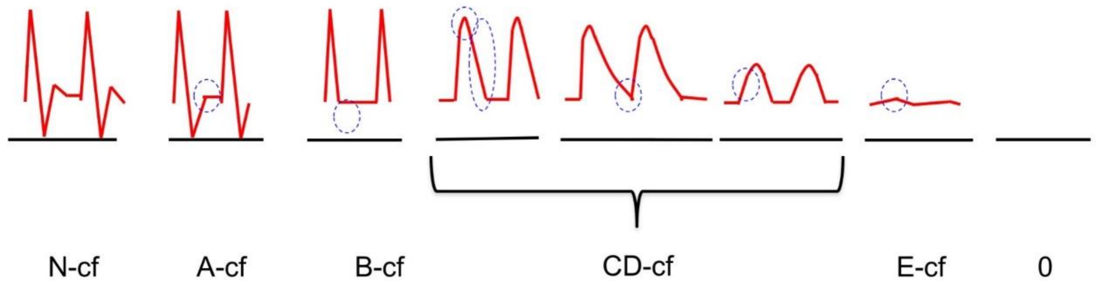

Classification of Saint Bonnet for false aneurysm flow

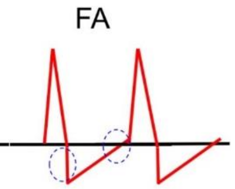

In some cases, the flow may not correspond to any flow in the classification, in which case the letter U should be used.

11

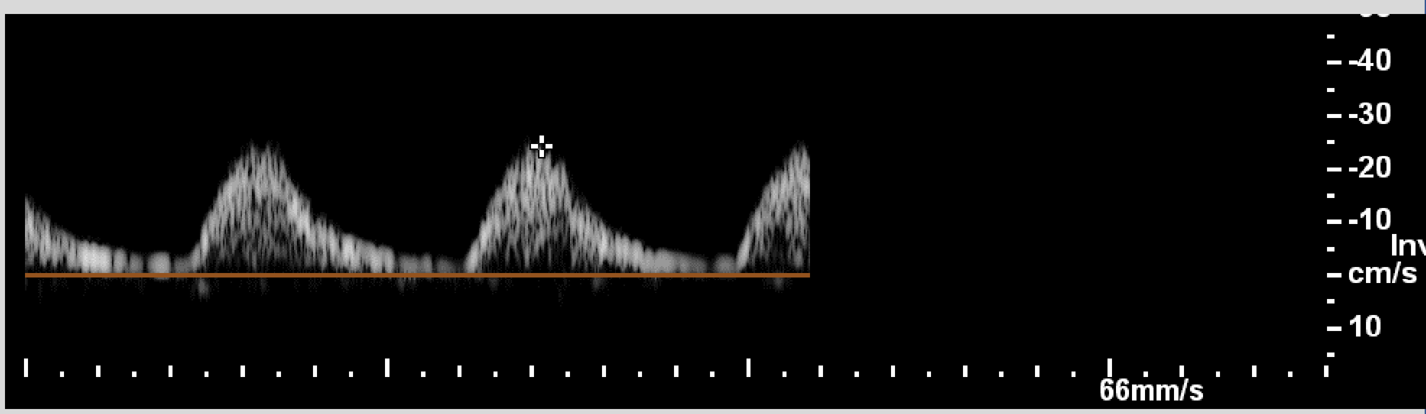

Can you describe this doppler waveform using the classification on the left side of the screen (Saint Bonnet).

Classification of Saint Bonnet without continuous flow

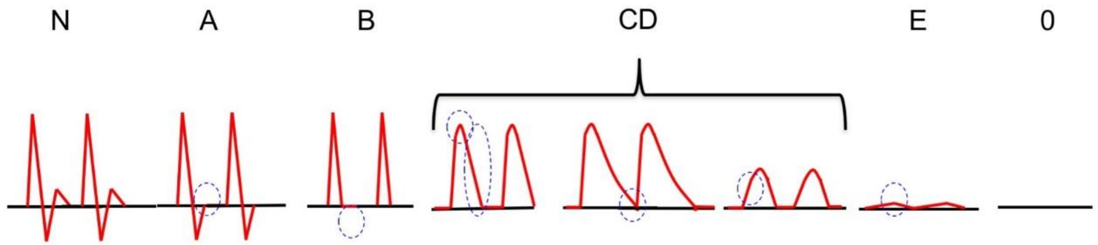

Classification of Saint Bonnet with continuous flow

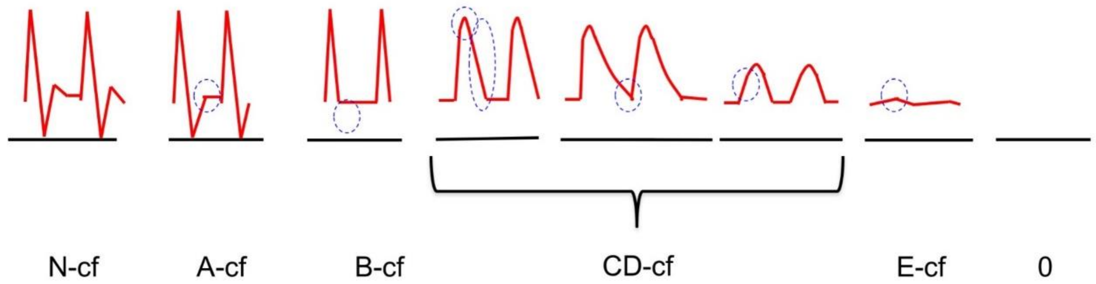

Classification of Saint Bonnet for false aneurysm flow

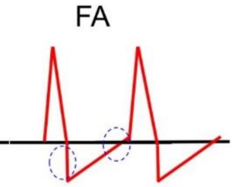

In some cases, the flow may not correspond to any flow in the classification, in which case the letter U should be used.

12

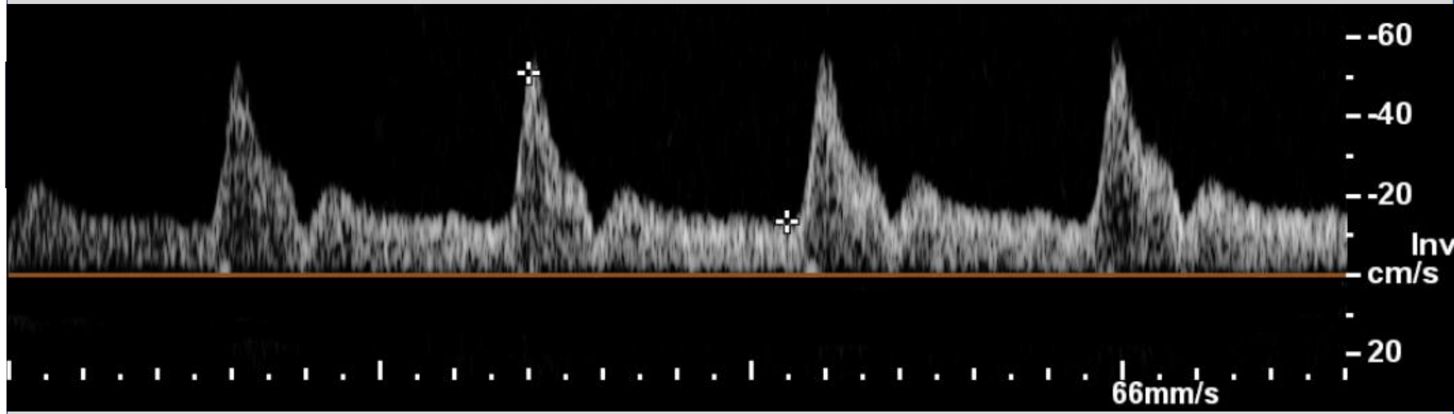

Can you describe this doppler waveform using the classification on the left side of the screen (Saint Bonnet).

Classification of Saint Bonnet without continuous flow

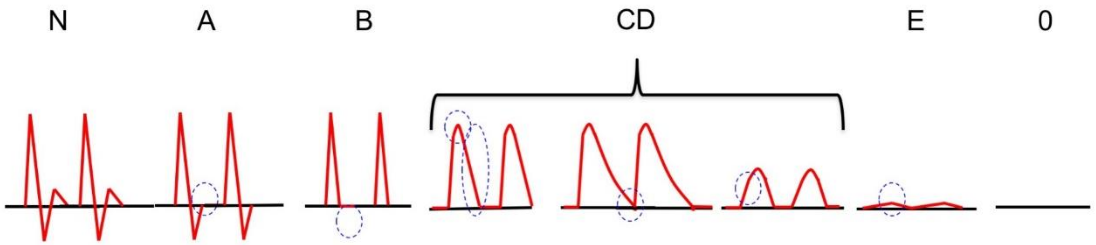

Classification of Saint Bonnet with continuous flow

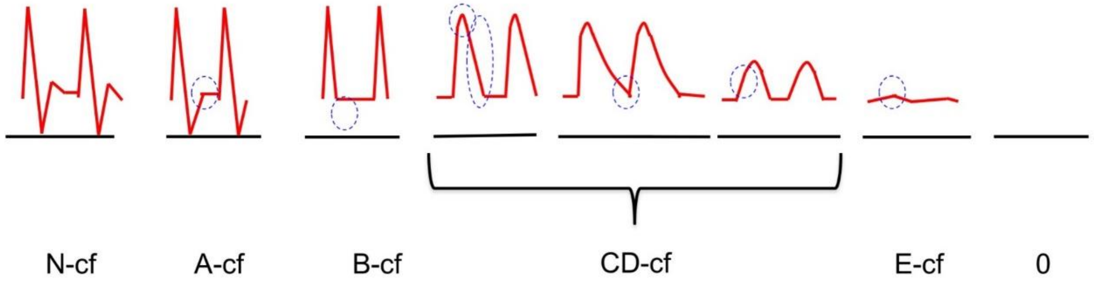

Classification of Saint Bonnet for false aneurysm flow

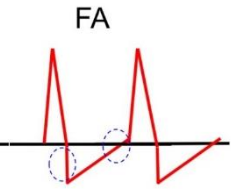

In some cases, the flow may not correspond to any flow in the classification, in which case the letter U should be used.

13

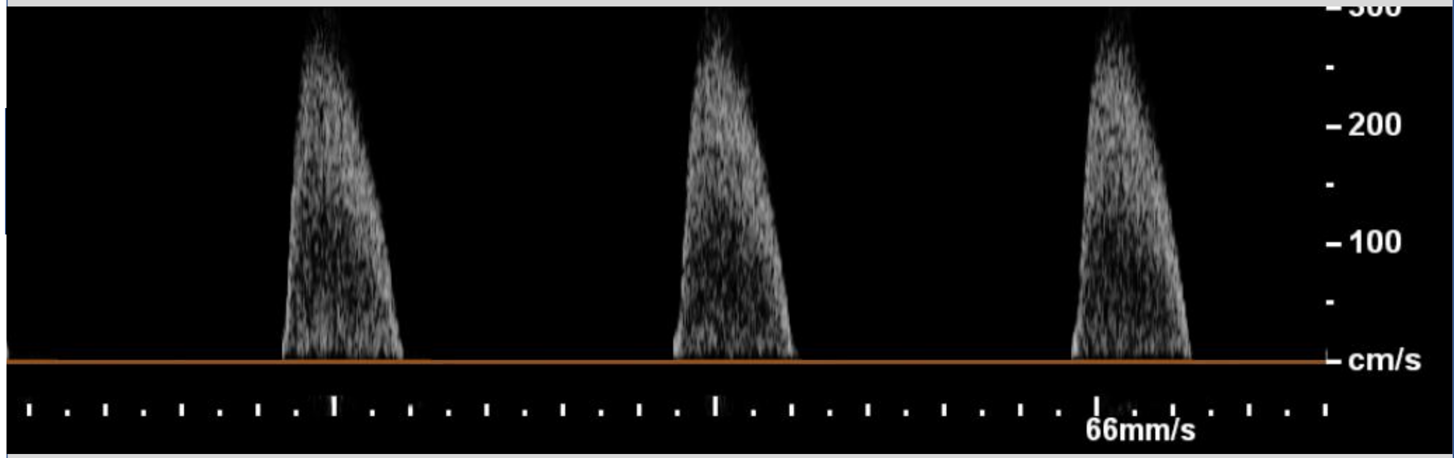

Can you describe this doppler waveform using the classification on the left side of the screen (Saint Bonnet).

Classification of Saint Bonnet without continuous flow

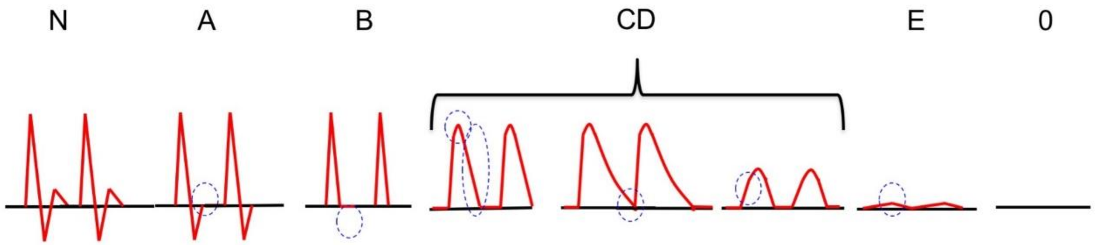

Classification of Saint Bonnet with continuous flow

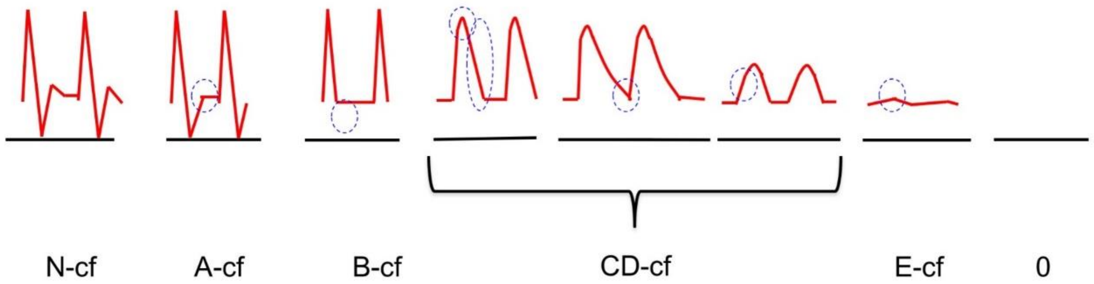

Classification of Saint Bonnet for false aneurysm flow

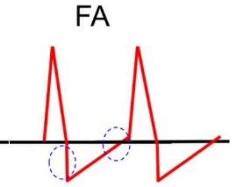

In some cases, the flow may not correspond to any flow in the classification, in which case the letter U should be used.

14

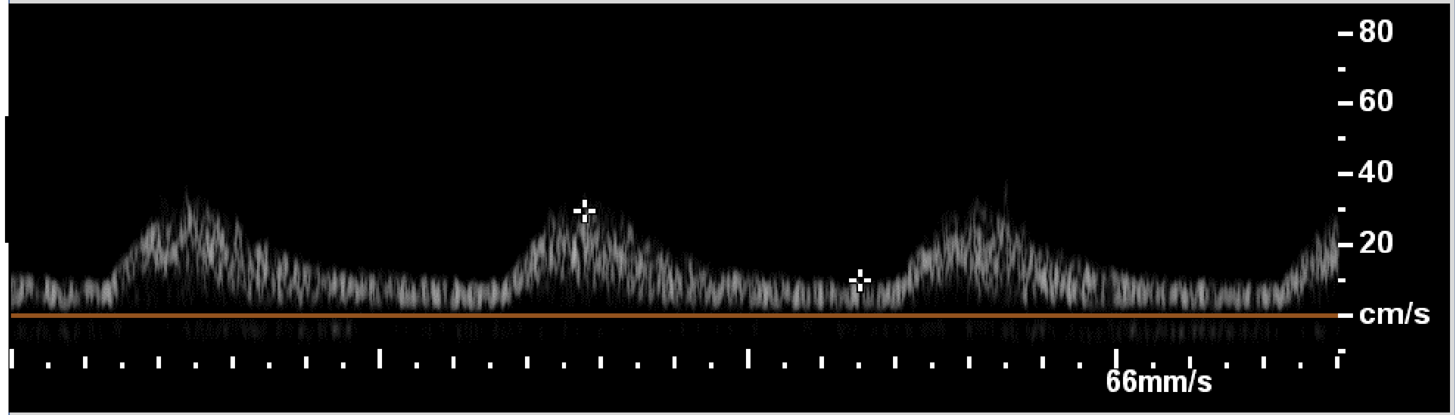

Can you describe this doppler waveform using the classification on the left side of the screen (Saint Bonnet).

Classification of Saint Bonnet without continuous flow

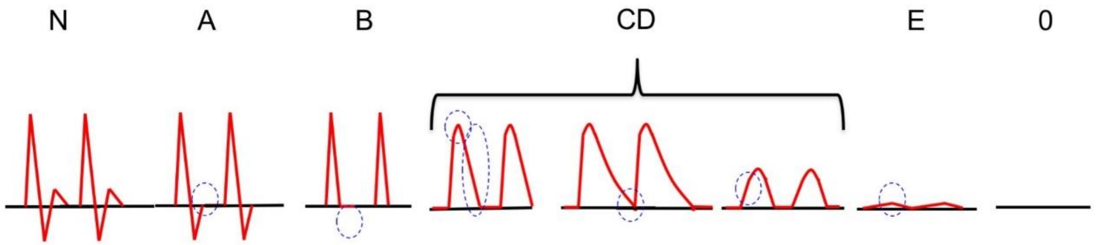

Classification of Saint Bonnet with continuous flow

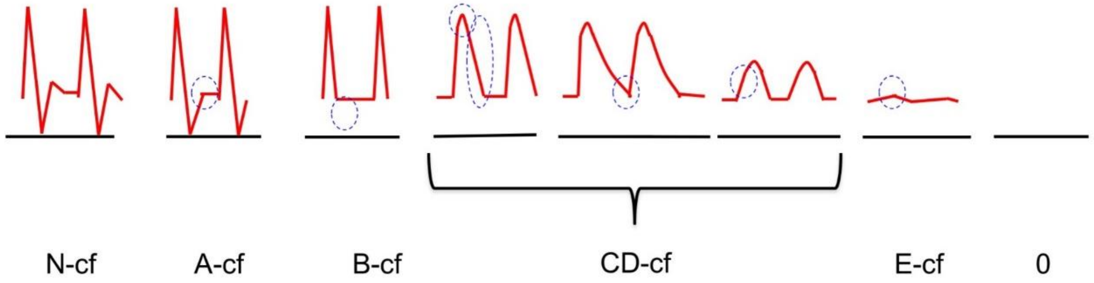

Classification of Saint Bonnet for false aneurysm flow

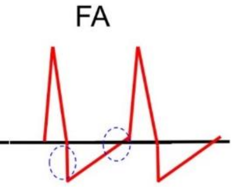

In some cases, the flow may not correspond to any flow in the classification, in which case the letter U should be used.

15

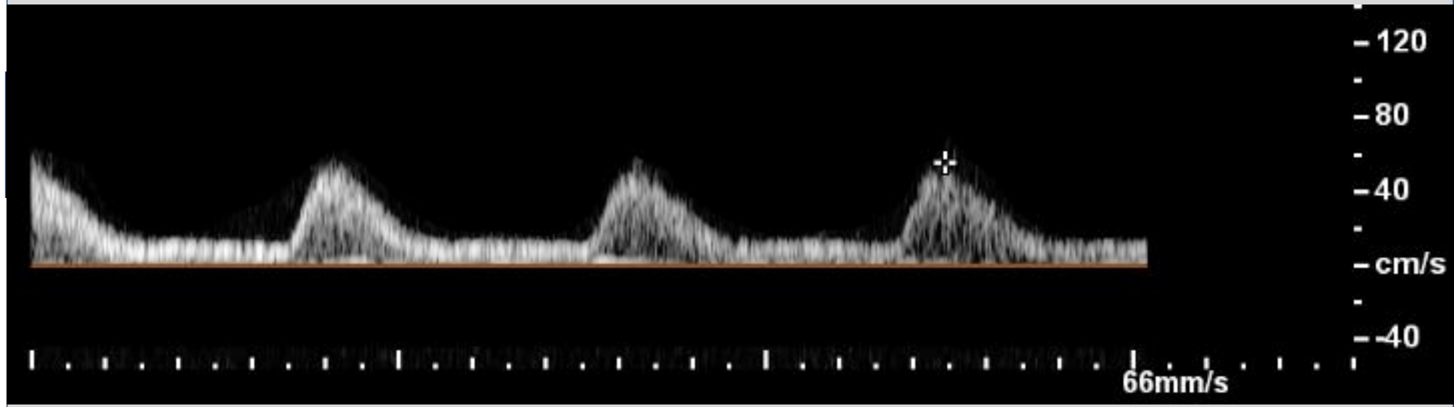

Can you describe this doppler waveform using the classification on the left side of the screen (Saint Bonnet).

Classification of Saint Bonnet without continuous flow

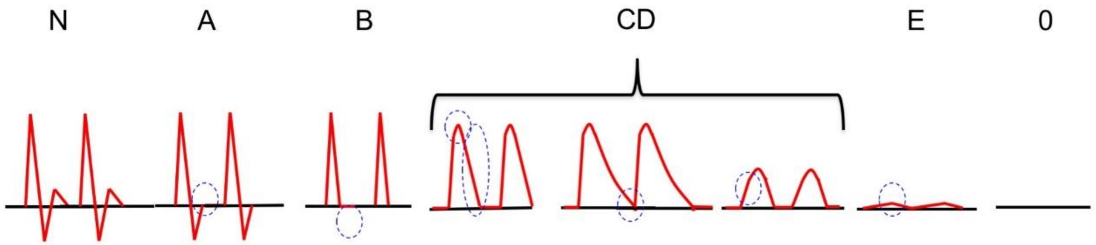

Classification of Saint Bonnet with continuous flow

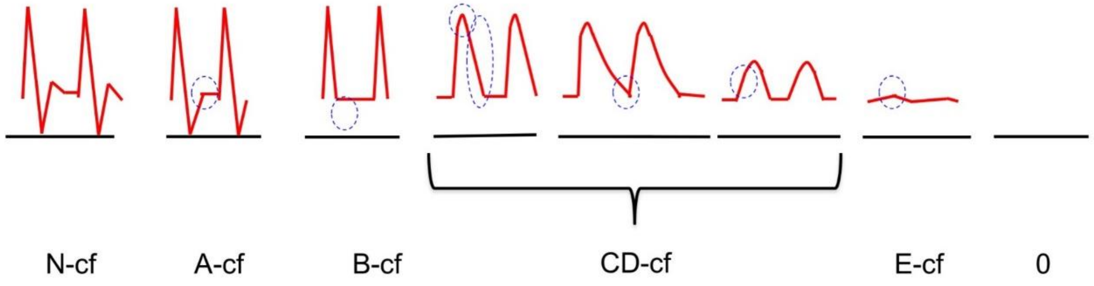

Classification of Saint Bonnet for false aneurysm flow

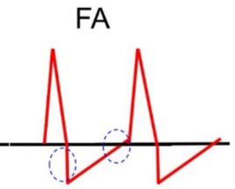

In some cases, the flow may not correspond to any flow in the classification, in which case the letter U should be used.

16

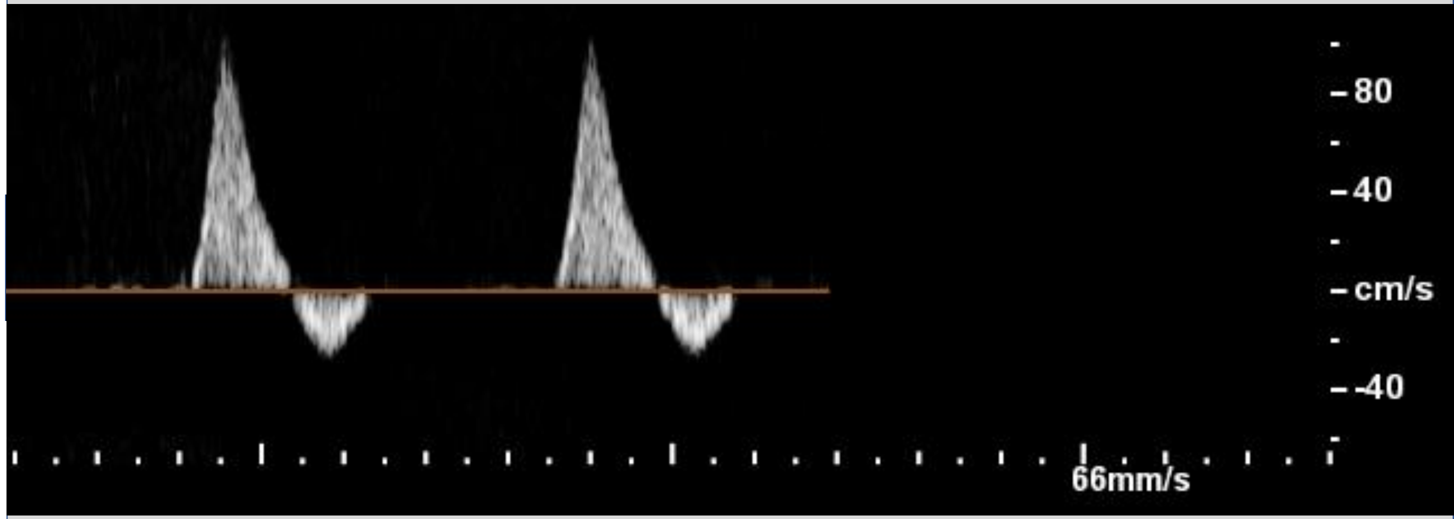

Can you describe this doppler waveform using the classification on the left side of the screen (Saint Bonnet).

Classification of Saint Bonnet without continuous flow

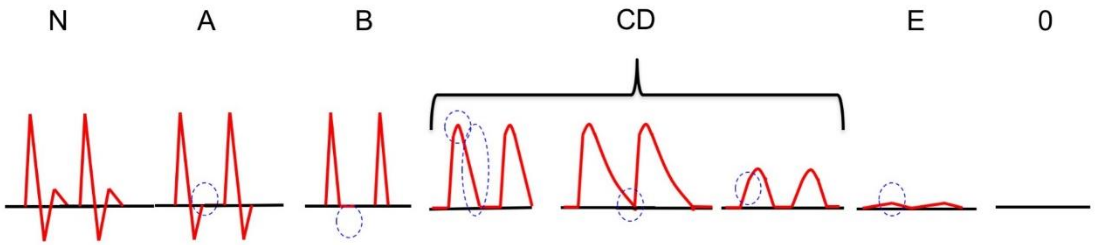

Classification of Saint Bonnet with continuous flow

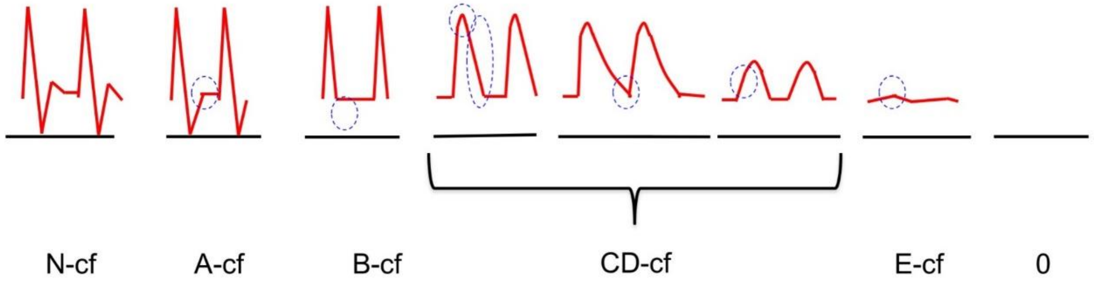

Classification of Saint Bonnet for false aneurysm flow

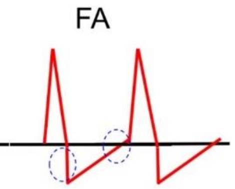

In some cases, the flow may not correspond to any flow in the classification, in which case the letter U should be used.

17

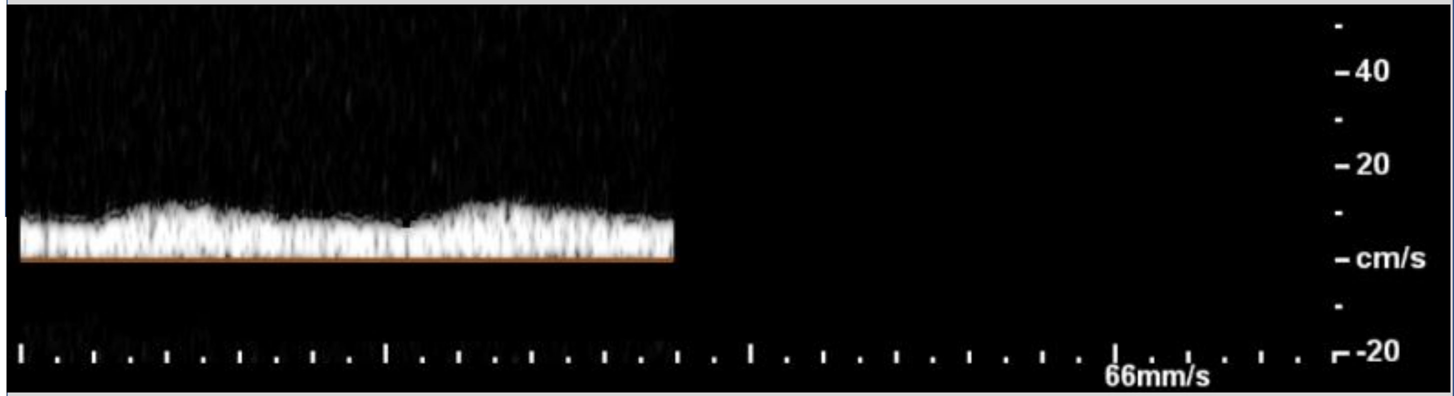

Can you describe this doppler waveform using the classification on the left side of the screen (Saint Bonnet).

Classification of Saint Bonnet without continuous flow

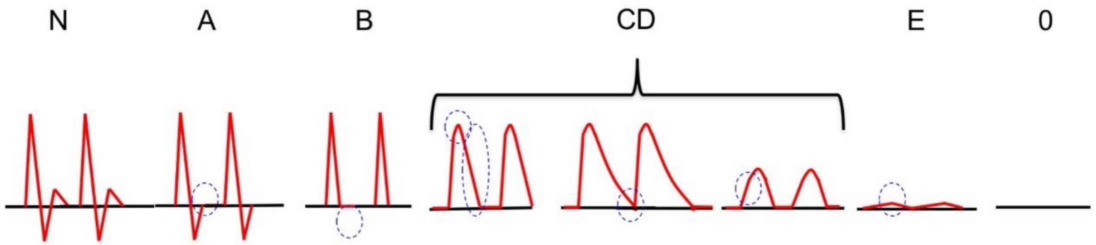

Classification of Saint Bonnet with continuous flow

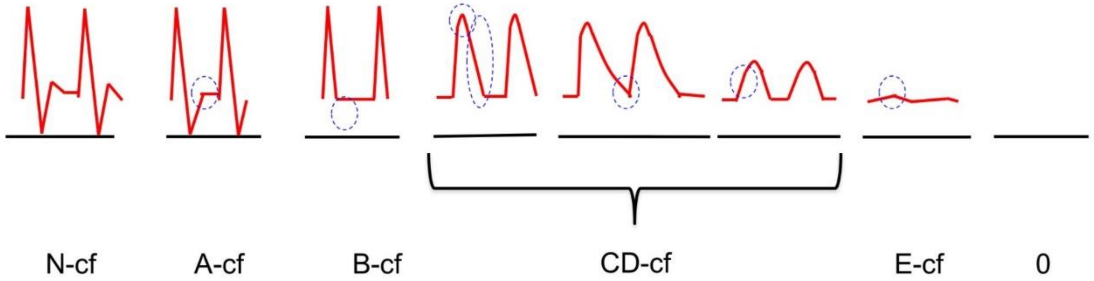

Classification of Saint Bonnet for false aneurysm flow

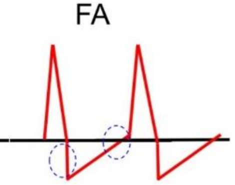

In some cases, the flow may not correspond to any flow in the classification, in which case the letter U should be used.

18

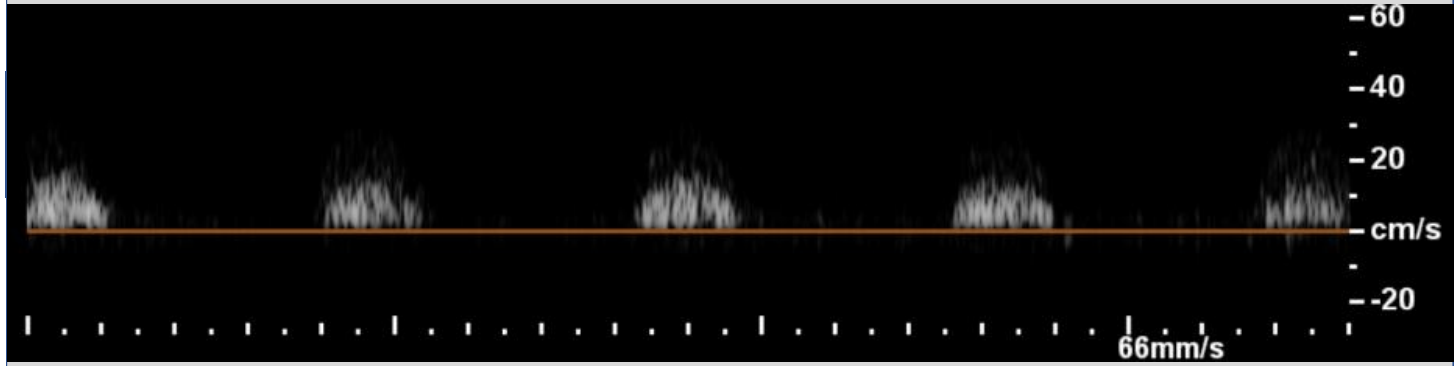

Can you describe this doppler waveform using the classification on the left side of the screen (Saint Bonnet).

Classification of Saint Bonnet without continuous flow

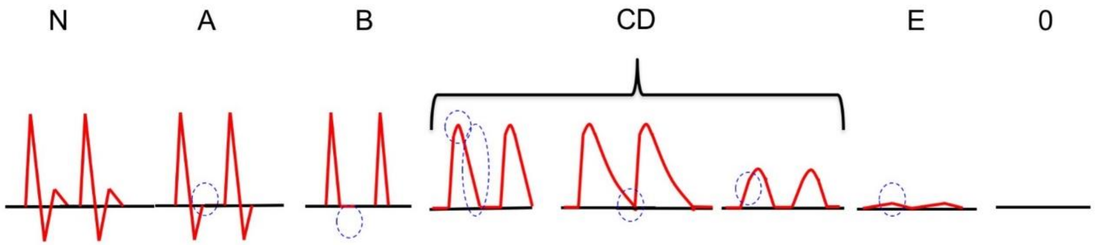

Classification of Saint Bonnet with continuous flow

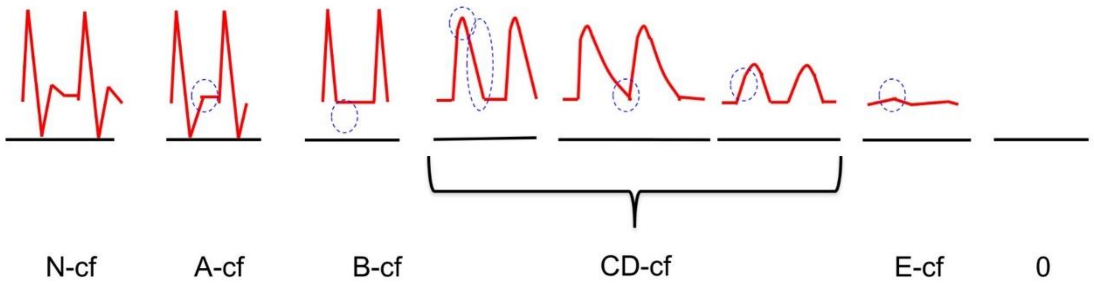

Classification of Saint Bonnet for false aneurysm flow

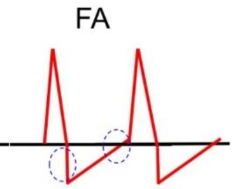

In some cases, the flow may not correspond to any flow in the classification, in which case the letter U should be used.

19

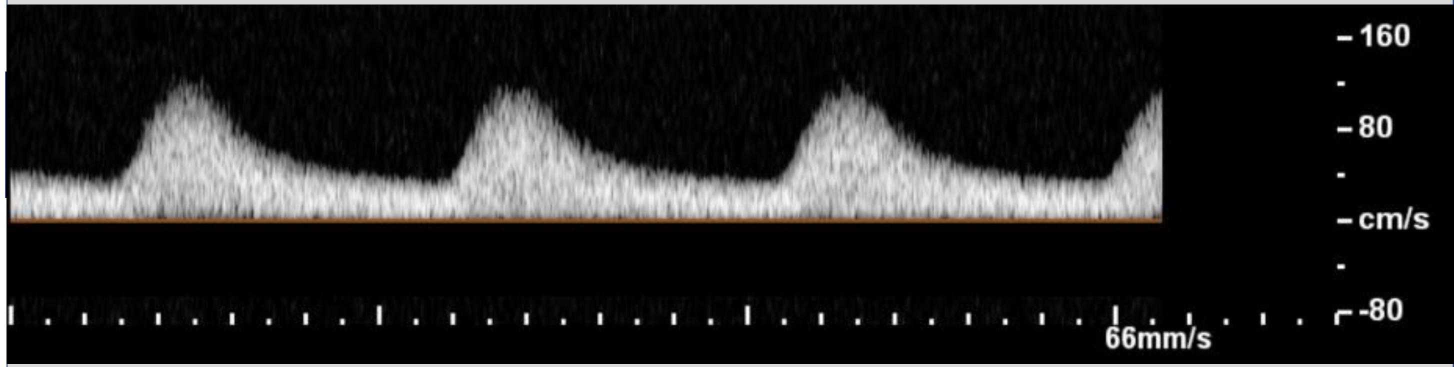

Can you describe this doppler waveform using the classification on the left side of the screen (Saint Bonnet).

Classification of Saint Bonnet without continuous flow

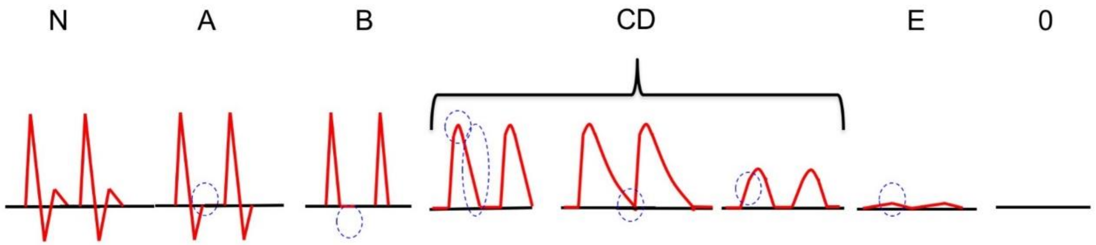

Classification of Saint Bonnet with continuous flow

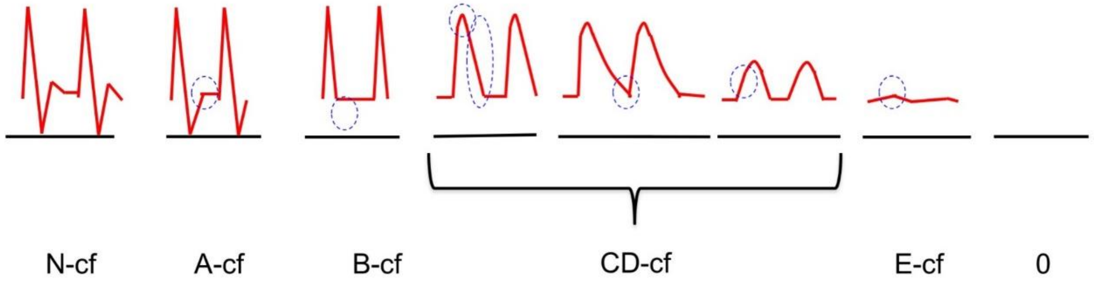

Classification of Saint Bonnet for false aneurysm flow

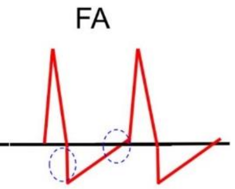

In some cases, the flow may not correspond to any flow in the classification, in which case the letter U should be used.

20

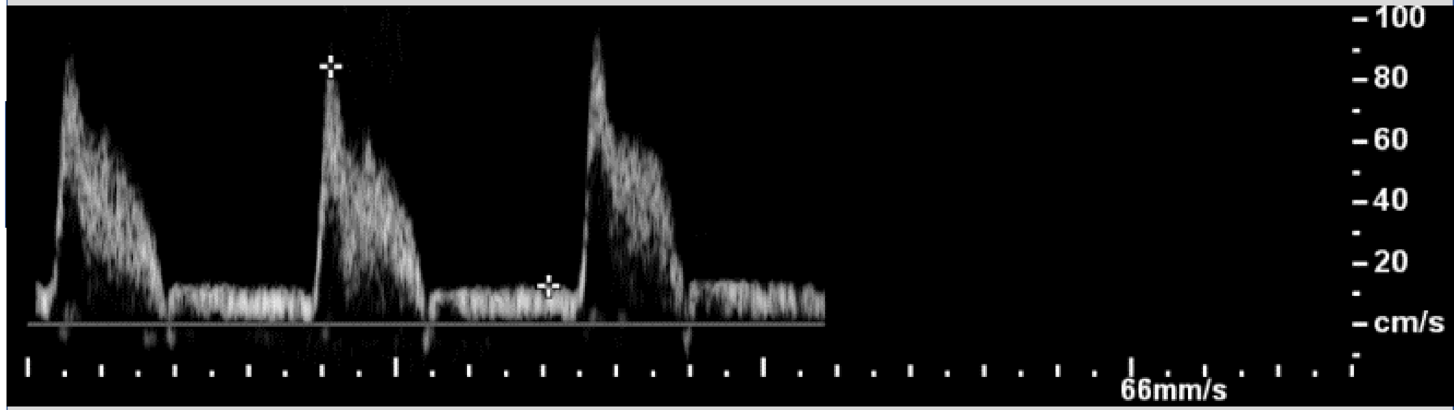

Can you describe this doppler waveform using the classification on the left side of the screen (Saint Bonnet).

Thanks for your participation
